# PGRL2 triggers degradation of PGR5 in the absence of PGRL1

**DOI:** 10.1038/s41467-021-24107-7

**Published:** 2021-06-24

**Authors:** Thilo Rühle, Marcel Dann, Bennet Reiter, Danja Schünemann, Belen Naranjo, Jan-Ferdinand Penzler, Tatjana Kleine, Dario Leister

**Affiliations:** 1 Plant Molecular Biology, Faculty of Biology, Ludwig-Maximilians University Munich, Planegg-Martinsried, Germany; 2 Molecular Biology of Plant Organelles, Ruhr University Bochum, Bochum, Germany

**Keywords:** Chloroplasts, Photosystem I

## Abstract

In plants, inactivation of either of the thylakoid proteins PGR5 and PGRL1 impairs cyclic electron flow (CEF) around photosystem I. Because PGR5 is unstable in the absence of the redox-active PGRL1, but not vice versa, PGRL1 is thought to be essential for CEF. However, we show here that inactivation of PGRL2, a distant homolog of PGRL1, relieves the need for PGRL1 itself. Conversely, high levels of PGRL2 destabilize PGR5 even when PGRL1 is present. In the absence of both PGRL1 and PGRL2, PGR5 alters thylakoid electron flow and impairs plant growth. Consequently, PGR5 can operate in CEF on its own, and is the target of the CEF inhibitor antimycin A, but its activity must be modulated by PGRL1. We conclude that PGRL1 channels PGR5 activity, and that PGRL2 triggers the degradation of PGR5 when the latter cannot productively interact with PGRL1.

## Abstract (web summary)

It is currently thought that the thylakoid proteins PGRL1 and PGR5 form a complex to mediate cyclic electron flow (CEF) around photosystem I. Here the authors show that CEF can in fact be mediated by PGR5 alone and that PGRL1 and the homologous PGRL2 modify the process by modulating PGR5 activity and stability.

## Introduction

In photosynthesis, linear electron flow (LEF) involves photosystems I (PSI) and II (PSII), together with the cytochrome (cyt) *b*_*6*_*f* complex, whereas PSII is dispensable for cyclic EF (CEF). LEF generates NADPH, and creates a trans-thylakoid proton gradient that is essential for ATP synthesis and the induction of non-photochemical quenching (NPQ), while CEF contributes only to the proton gradient^1–4^. During LEF, electrons received by ferredoxin (Fd) from PSI are transferred to NADP^+^ via Fd-NADP^+^ reductase (FNR). However, under CEF conditions, they are either diverted to the NADH dehydrogenase-like complex (NDH) in the antimycin A (AA)-insensitive CEF pathway^5,6^, or passed on to an alternative pathway designated “AA-sensitive CEF”^4,7–9^. Several scenarios for AA-sensitive CEF are under discussion^10–15^. One of them postulates that, in plants, plastoquinone (PQ) is reduced by a Fd-PQ reductase (FQR)^16^, and the identification of the thylakoid proteins PGR5 and PGRL1^17,18^ appears to support this idea.

PGR5 was discovered in a genetic screen for *Arabidopsis thaliana* mutants with an altered trans-thylakoid proton gradient^17^. In addition to a decrease in both steady-state and transiently induced NPQ, *pgr5* mutants display enhanced PSI photoinhibition and die when exposed to fluctuating light (FL) levels^17,19^. However, the pleiotropic nature of the *pgr5* phenotype, together with the lack of obvious redox-active moieties in PGR5, has prompted alternative suggestions for the primary function of PGR5, including a role in the regulation of LEF^19,20^. Some of the apparent shortcomings of PGR5 as the sole mediator of CEF were mitigated when PGRL1 was identified, as its inactivation gives rise to a *pgr5*-like phenotype^18^. In vitro, PGRL1 was reported to accept electrons from Fd in a PGR5-dependent manner, and reduces quinones in an AA-sensitive fashion^21^. Moreover, PGRL1 contains several redox-active cysteine residues and a Fe-containing cofactor^21^, and redox regulation of PGRL1 activity involves PGR5- and thioredoxin m4-dependent formation of disulfide bridges^21–23^. PGRL1 interacts at least transiently with cyt *b*_*6*_*f* and PSI^18^ and its loss drastically decreases the abundance of PGR5 in *A. thaliana* but not vice versa^18,24,25^. In addition, both proteins have characteristics that are compatible with the AA sensitivity of the FQR pathway^21,26^. Hence, the current view is that (i) PGRL1 serves as a membrane anchor for PGR5, and (ii) the two proteins together constitute the FQR^2,21^. PGRL1 is several-fold more abundant than PGR5^21^, suggesting that PGR5 might be limiting for CEF—and indeed CEF is enhanced upon overexpression of PGR5^27,28^. More recently, Arabidopsis PGR5 and PGRL1 have been shown to drive CEF effectively in the cyanobacterium *Synechocystis* sp. PCC6803 (hereafter *Synechocystis*), without requiring additional plant-specific proteins^29^.

In this study, we present results that force a radical revision of the conventional view of the function of PGR5 and its relationship to PGRL1. We demonstrate that PGR5 is the central element in the formation of the trans-thylakoid proton gradient during AA-sensitive CEF, and that PGRL1 stabilizes the protein and limits its activity to prevent negative side-effects on thylakoid electron flow and plant growth. We also prove that PGRL1 is not the target of CEF inhibition by the chemical AA in vivo. In consequence, we present a model in which a distant homolog of PGRL1, which we designate as PGRL2, promotes the degradation of PGR5 when the latter’s interaction with PGRL1 is disrupted by inactivation of PGRL1, mutation of PGR5, or an excess of PGRL2.

## Results

### PGRL1 is not essential for plant survival under fluctuating light

**Fig. 1:**
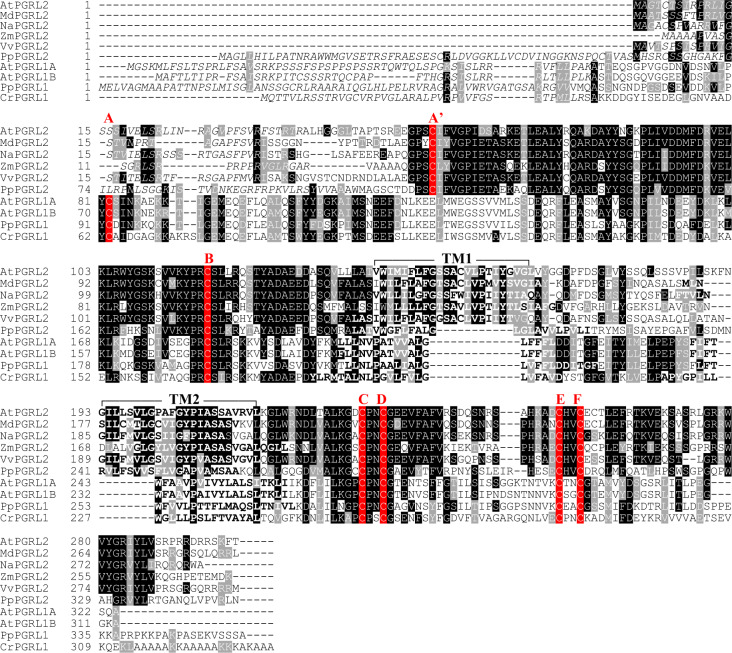
PGRL2 and PGRL1 share characteristic features. Multiple sequence alignment of PGRL1 and PGRL2 family members. Conserved cysteines are highlighted in alphabetical order. Aligned cysteines identified only in PGRL2 are depicted as **A’**. Predicted transit peptide sequences and transmembrane domains (see “Methods”) are shown in italic and bold letters, respectively. The two AtPGRL2 transmembrane domains are indicated by the overhead brackets marked “TM1” and “TM2”. Instances of sequence identity/similarity in at least 40% of the sequences are highlighted by black/gray shading. The accession numbers of the sequences are listed in Supplementary Table 1.

**Fig. 2:**
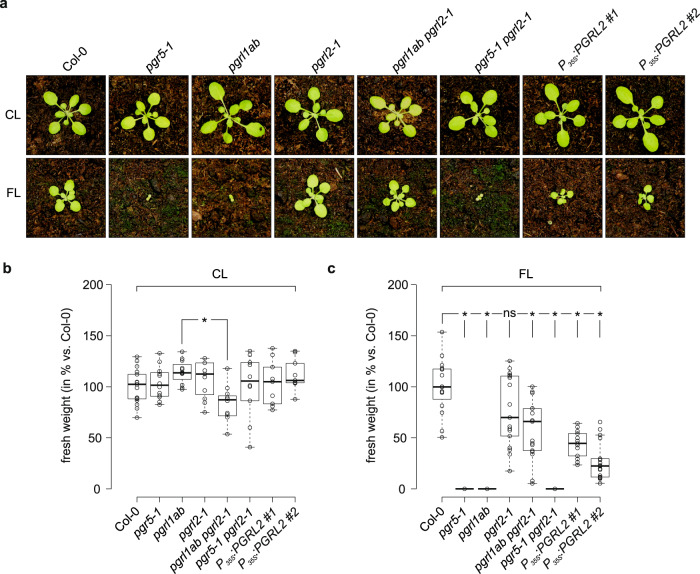
Plants can survive under fluctuating light conditions when both PGRL1 and PGRL2 are absent. **a** Images of 5-week-old WT (Col-0) plants, mutants (*pgr5-1*, *pgrl1ab*, *pgrl2-1*, *pgrl1ab pgrl2-1*, and *pgr5-1 pgrl2-1*) and two independent *P*_*35S*_*:PGRL2* (Col-0 background) lines grown under either 12 h fluctuating light (cycles of 5 min at 50 μmol photons m^−2^ s^−1^/1 min at 500 μmol photons m^−2^ s^−1^) /12 h dark cycles (FL) or 12 h constant light (100 μmol photons m^−2^ s^−1^) /12 h dark cycles (CL). **b**, **c** Fresh weight (in % to the average of Col-0) of rosette leaves was determined under CL (**b**) and FL (**c**). Data points of the eight genotypes are plotted as open circles (*n* = 17, 12, 12, 11, 12, 10, 9, 9 in **b**; *n* = 15, 17, 17, 17, 17, 17, 13, 17 in **c**). The horizontal lines in **b**, **c** represent the median; boxes indicate the 25th and 75th percentiles. Whiskers extend 1.5× the interquartile range, outliers are represented as dots. For statistical analyses in panel **b**, **c**, the non-parametric Kruskal–Wallis test was performed, followed by pairwise Dunn’s tests. The *p*-values were adjusted on an experiment level using the Benjamini–Hochberg method. Statistically significant differences are marked with asterisks (*p ≤ 0.05, ns, not statistically significant). *P*-value in panel b: 1.9 × 10^−3^. *P*-values in **c** (order as displayed): 3.7 × 10^−11^, 1.1 × 10^−10^, 0.2, 4.5 × 10^−2^, 5.6 × 10^−11^, 1.2 × 10^−2^ and 3.7 × 10^−4^.

*Arabidopsis thaliana* expresses two PGRL1 isoforms (A and B), and the protein At5g59400 has previously been identified as their closest paralog^30^, sharing 19%/34% identity/similarity with PGRL1A and B. We revisited these sequence comparisons and found that this PGRL1 paralog, which we designate as PGRL2, is present in plants and in *Micromonas* sp. and other green algae, but not in red algae (Supplementary Fig. 1, Supplementary Table 1). Like PGRL1, PGRL2 has two transmembrane domains and contains five of the six conserved cysteine residues found in PGRL1 (Fig. 1). We isolated a mutant line that lacks *PGRL2* expression, complemented the mutation *pgrl1ab* *pgrl2-1* with the WT *PGRL2* gene (Supplementary Fig. 2a–c), and generated the triple mutant *pgrl1ab pgrl2-1* and the double mutant *pgr5-1 pgrl2-1*. Intriguingly, *pgrl1ab pgrl2-1* plants survive under fluctuating light (FL) conditions (cycles of 5 min at 50 μmol photons m^−2^ s^−1^ and 1 min at 500 μmol photons m^−2^ s^−1^ during the day), unlike *pgr5-1, pgrl1ab* and *pgr5-1 pgrl2-1* plants (Fig. 2a). Under 12 h light/12 h dark cycles (CL conditions), *pgrl1ab pgrl2-1* plants grew significantly more slowly than the *pgrl1ab* line (Fig. 2b). Lines overexpressing the *PGRL2* gene (Supplementary Fig. 2d) barely survived under FL conditions (Fig. 2c) and accordingly accumulated far less fresh weight relative to the other genotypes that were viable under FL conditions (Col-0, *pgrl2-1*, and *pgrl1ab pgrl2-1*).

These results show that inactivation of *PGRL2* enables *pgrl1ab* plants to remain viable under FL conditions, albeit at the cost of reduced growth under CL conditions. However, the *pgrl2-1* mutation cannot suppress *pgr5-1* lethality under FL. Moreover, increasing the amount of PGRL2 suppresses plant growth under FL, but not CL conditions. Because viability under FL conditions is thought to require the function of PGR5, this suggests that PGR5 could function in the absence of PGRL1, and that PGRL2 might have a negative effect on the function.

### PGR5 can function in CEF in the absence of PGRL1

**Fig. 3:**
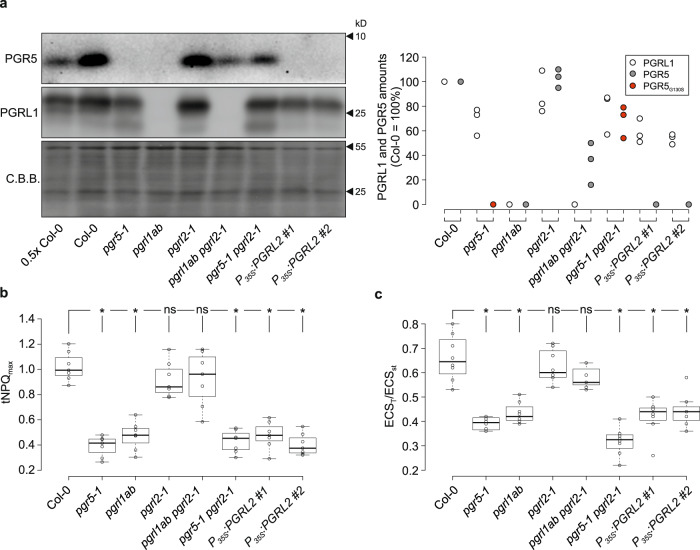
PGR5 can mediate CEF in the absence of PGRL1. **a** Aliquots of leaf proteins prepared from CL plants shown in Fig. 2 were fractionated by SDS-PAGE and subjected to immunoblotting using PGR5- or PGRL1-specific antibodies. PVDF membranes were stained with Coomassie brilliant blue (C.B.B.) as loading control. Quantification of PGRL1, PGR5, and PGR5_G130S_ amounts from three experiments (WT = 100) are shown as dot plots on the right. **b** Maximal transient NPQ (tNPQ_max_) values determined during dark-to-light (110 µmol photons m^−2^ s^−1^) transitions (see “Methods”). Data points are shown as open circles (*n* = 7). **c** Electrochromic shift (ECS) was measured to assess the proton motive force (PMF) (see “Methods”). Plants were exposed to high light intensities (340 µmol photons m^−2^ s^−1^) for 15 min, and the change in absorbance at 515 nm (ECS_T_) was recorded in dark interval relaxation kinetics. ECS_T_ values were normalized to the ECS_st_, the absorbance change at 515 nm evoked by a single turnover flash prior to exposure to high light levels. Data points are shown as open circles (*n* = 8, 8, 8, 8, 7, 8, 8, 8). The horizontal lines in **b**, **c** represent the median, and boxes indicate the 25th and 75th percentiles. Whiskers extend 1.5× the interquartile range, outliers are represented as dots. For statistical analyses in (**b**, **c**), the non-parametric Kruskal–Wallis test was performed, followed by pairwise Dunn’s tests. The *p*-values were adjusted on an experiment level using the Benjamini–Hochberg method. Statistically significant differences are marked with asterisks (*p ≤ 0.05, ns, not statistically significant). *P*-values in **b** (order as displayed): 6.8 × 10^−4^, 3.6 × 10^−3^, 0.4, 0.4, 1.4 × 10^−3^, 3.6 × 10^−3^ and 9.1 × 10^−4^. *P*-values in **c** (order as displayed): 1.8 × 10^−4^, 4.7 × 10^−3^, 0.5, 0.4, 3.8 × 10^−6^, 4.5 × 10^−3^ and 7.2 × 10^−3^.

When levels of PGR5 and PGRL1 were quantified in the different genotypes by Western blot analysis, PGR5 was found to accumulate to about 35% of WT levels in *pgrl1ab pgrl2-1* plants (Fig. 3a). Thus, in the absence of PGRL2, PGRL1 is no longer essential for PGR5 accumulation. Moreover, the inactivation of PGRL2 in *pgr5-1 pgrl2-1* plants boosts steady-state amounts of the mutant PGR5 protein (PGR5_G130S_, in which the glycine at position 130 in the WT protein is replaced by a serine^17^) to about 70% of wild-type (WT) PGR5 levels (Fig. 3a). In *pgrl2-1* plants, levels of PGR5 were not affected, whereas overexpression of PGRL2 rendered the protein undetectable (Fig. 3a). The effective loss of PGR5 in PGRL2 overexpressors raises the question of how these plants can still survive under FL (see Fig. 2a), in contrast to the other genotypes that lack a functional PGR5 protein (*pgrl1ab* and *pgr5-1* plants). To clarify this issue, we also investigated dark-incubated PGRL2 overexpressors and found residual levels of PGR5 (equivalent to 15-25% of WT levels) (Supplementary Fig. 2e). This indicates that the destruction of PGR5 in the presence of excess PGRL2 is light dependent, and suggests that the levels of PGR5 synthesized in the dark suffice to maintain viability under FL conditions.

Taken together, these results imply that the PGRL2 protein negatively affects the accumulation of PGR5, in particular when PGRL2 is present in excess (as in PGRL2 overexpressors) or when PGRL1 is absent (as in *pgrl1ab*). PGRL2 appears also to be involved in destabilizing the PGR5_G130S_ mutant encoded by *pgr5-1 in planta*, because removal of PGRL2 restores accumulation of the protein. This might be attributable to the lower levels of PGRL1 found in *pgr5-1* plants and/or because the mutated PGR5 is more susceptible to PGRL2-dependent degradation than the WT protein.

Next, we measured the kinetics of transient NPQ induction and the electrochromic shifts (ECS) that occur during charge-transfer processes as proxies for CEF activity (Fig. 3b, c). The extent of transient NPQ induction upon a dark-to-light shift serves as a measure of CEF activity^17^ and in this assay the maximum transient NPQ (tNPQ_max_) during the induction-recovery curve is drastically reduced in *pgr5-1* and *pgrl1ab* plants, as well as in the *pgr5-1 pgrl2-1*, and PGRL2-overexpressing plants, compared to the WT control (Fig. 3b, Supplementary Fig. 3). In *pgrl1ab pgrl2-1* plants, WT-like tNPQ_max_ values were recorded, indicating that the levels of PGR5 (~35% of WT) detected in this line (see Fig. 3a) suffice to mediate WT-like CEF in the absence of both PGRL1 and PGRL2. The proton motive force (PMF) across the thylakoid membrane arises from LEF and CEF, and can be measured by electrochromic shift (ECS) analysis. To this end, ECS_T_/ECS_st_, the ratio of the total light-dark amplitude of ECS (ECS_T_) and the change in absorbance at 515 nm induced by an initial single turnover flash (ECS_st_), was determined as described previously^31^ (see “Methods”). The ECS_T_/ECS_st_ pattern among the different genotypes was similar to that observed in the tNPQ_max_ analysis (Fig. 3c). The ECS_T_/ECS_st_ values measured in WT (Col-0), *pgrl2-1*, and *pgrl1ab pgrl2-1* were all in the same range, whereas *pgr5-1*, *pgrl1ab*, and the PGRL2 overexpressors showed significantly lower values, with *pgr5-1 pgrl2-1* displaying the lowest average value.

### PGR5 enhances CEF in the absence of PGRL1

**Fig. 4:**
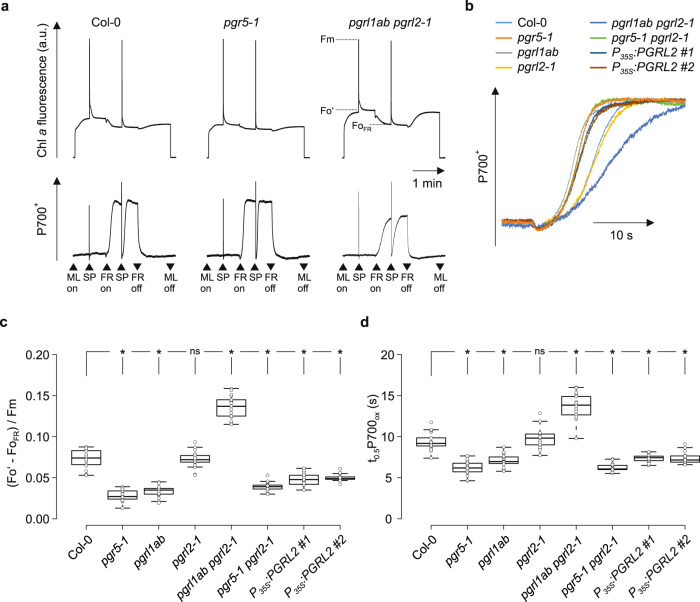
Plastoquinone reduction and PSI electron transport are altered in the absence of PGRL1 and PGRL2 under very low light. **a** Chl *a* fluorescence and P700 oxidation kinetics were recorded simultaneously with a Dual-PAM system as described^27^. After switching on measuring light (ML, 1 µmol photons m^−2^ s^−1^) and applying an initial saturating pulse (SP), leaves adapted to dark for 1 h before were exposed to far-red light (FR) for 60 s. The FR light treatment was interrupted by a second SP after 30 s. **b** Excerpt from the P700 oxidation kinetics during FR exposure. Graphs were normalized to the minimal and maximal P700^+^ levels which were recorded in between the first and second SP. **c** Quantification of the Chl *a* fluorescence drop shortly after FR treatment. The difference of the Chl *a* fluorescence ground state in ML (Fo’) and the minimal Chl *a* fluorescence level (Fo_FR_) during FR treatment was normalized to the maximal Chl fluorescence (Fm). **d** Quantification of P700^+^ oxidation half time t_0.5_P700_ox_ after FR light exposure. Seventeen replicates for every genotype were measured. The horizontal lines in **c**, **d** represent the median, and boxes indicate the 25th and 75th percentiles. Whiskers extend 1.5× the interquartile range, outliers are represented as dots. For statistical analyses in (**c**, **d**), the non-parametric Kruskal–Wallis test was performed, followed by pairwise Dunn’s tests. The *p*-values were adjusted on an experiment level using the Benjamini–Hochberg method. Statistically significant differences are marked with asterisks (*p ≤ 0.05, ns, not statistically significant). *P*-values in **c** (order as displayed): 6.5 × 10^−10^, 5.2 × 10^−8^, 0.5, 0.04, 8.1 × 10^−6^, 5.1 × 10^−3^ and 1.2 × 10^−2^. *P*-values in **d** (order as displayed): 1.0 × 10^−7^, 2.9 × 10^−4^, 0.4, 0.03, 3.6 × 10^−8^, 3.5 × 10^−3^ and 2.8 × 10^−3^.

We further investigated CEF activity with an assay that allows to simultaneously examine plastoquinone reduction and P700 oxidation with a Dual-PAM system^27^ (Fig. 4). To this end, single attached leaves were exposed to a very low light intensity of 1 µmol photons m^−2^ s^−1^ to fuel minimal photosynthetic electron transport rates for CEF determination. The degree of plastoquinone reduction was determined by comparing the minimal chlorophyll fluorescence levels in the absence (Fo’) and presence (Fo_FR_) of far-red (FR) light (Fig. 4a), whereas PSI activity was evaluated by measuring P700 oxidation kinetics during FR exposure (Fig. 4b). The drop of Fo’ levels after FR light exposure was lower (Fig. 4c) and P700 oxidation half times (t_0.5_P700_ox_) were shorter (Fig. 4d) for *pgr5-1*, *pgrl1ab*, *pgr5-1 pgrl2-1*, and *P*_*35S*_*:PGRL2* lines compared to Col-0 and *pgrl2-1*. On the contrary, *pgrl1ab pgrl2-1* lines were characterized by a significantly higher Fo’ level (Fig. 4c) and slower P700 oxidation rates with respect to Col-0 and *pgrl2-1* (Fig. 4d). These data indicate that lines with no (or mutated) PGR5 were impaired in CEF activity, leading to lower plastoquinone reduction and faster P700 oxidation rates. In contrast to this, lack of both PGRL1 and PGRL2 in *pgrl1ab pgrl2-1* induced higher CEF rates and mimicked the phenotype observed for lines overexpressing *PGR5* with respect to plastoquinone reduction and P700 oxidation under minimal photosynthetic electron transport rates^27^.

Comparisons of the steady-state levels of PGR5 and PGRL1 with the data for tNPQ_max_, ECS_T_/ECS_st_, plastoquinone reduction, and P700 oxidation kinetics lead to a number of conclusions. (1) CEF requires PGR5 but not PGRL1. (2) Excess amounts of PGRL2 suppress both PGR5 accumulation and CEF, as seen in the PGRL2 overexpressors. (3) PGR5_G130S_ cannot mediate CEF activity even when present in amounts as high as 70% of WT PGR5 (in *pgr5-1 pgrl2-1*). In fact, the functionality of PGR5_G130S_ could not be unambiguously assessed previously, because it failed to accumulate either *in planta* (in the *pgr5-1* single mutant) or in our cyanobacterial testbed, whether expressed alone or together with PGRL1^29^. (4) PGR5-dependent CEF is controlled by PGRL1 and PGRL2, since their absence resulted in significantly higher plastoquinone reduction and slower P700 oxidation rates in *pgrl1ab pgrl2-1*. Strikingly, the ~35% of WT levels of PGR5 remaining in the *pgrl1ab pgrl2-1* mutant result in higher CEF activity than the ~100% in WT and *pgrl2-1* plants.

### Photosynthesis in low light is compromised if PGR5 accumulates in the absence of PGRL1

**Fig. 5:**
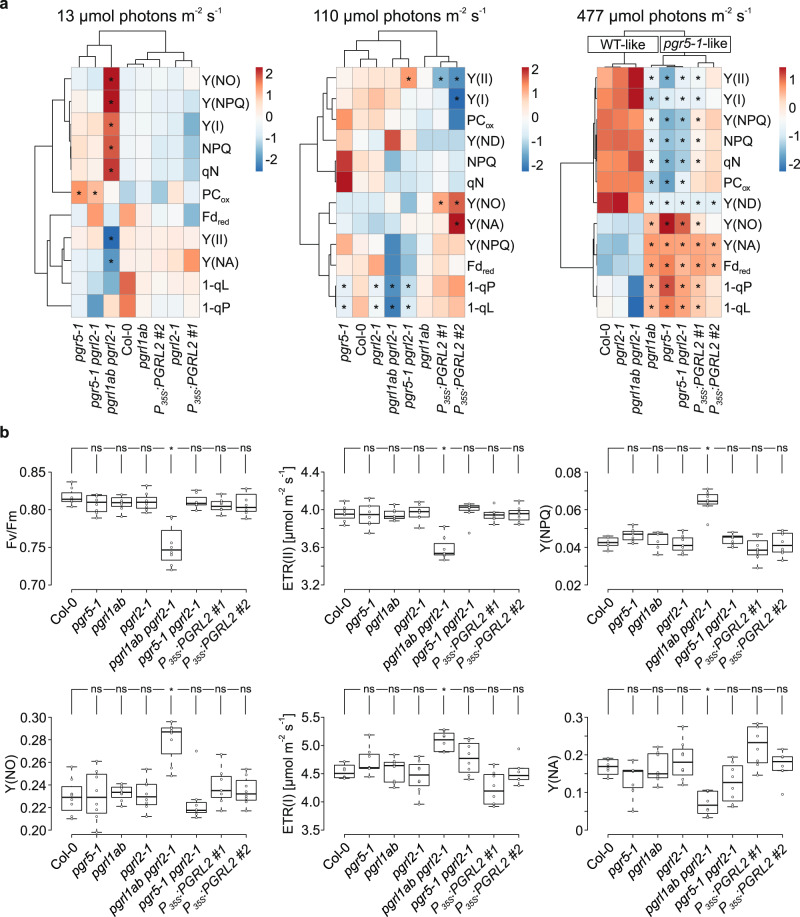
Thylakoid electron flow under different light intensities. **a** Hierarchically clustered heat map analyses of photosynthetic parameters (see “Methods”) determined under three different light intensities. Data represent mean values of eight (13 µmol photons m^−2^ s^−1^), seven (13 µmol photons m^−2^ s^-1^) and eight (477 µmol photons m^−2^ s^−1^) replicates, and were obtained from light induction/recovery measurements with a Dual-KLAS/NIR system (see “Methods”). Values were standardized according to the unit variance scaling method and are represented on a blue (low values) to red (high values) color scale. Rows and columns are clustered using correlation distance and average linkage. Statistically significant differences relative to the WT are indicated by the asterisks (*p ≤ 0.05). **b** Steady-state photosynthetic parameters (maximum quantum yield of PSII [Fv/Fm], electron transport rates through PSII [ETR(II)], regulated energy dissipation in PSII [Y(NPQ)], nonregulated energy dissipation in PSII [Y(NO)], electron transport rates through PSI [ETR(I)] and acceptor-site limitation of PSI [Y(NA)]) after 341s light induction at low light (13 µmol photons m^−2^ s^−1^). Plants were dark-adapted for 30 min before Fv/Fm determination. Eight replicates for every genotype were measured. The horizontal lines in panel b represent the median, and boxes indicate the 25th and 75th percentiles. Whiskers extend 1.5× the interquartile range, outliers are represented as dots. For statistical analyses in (**a**, **b**), the non-parametric Kruskal–Wallis test was performed, followed by pairwise Dunn’s tests. The *p*-values were adjusted on an experiment level using the Benjamini–Hochberg method. Statistically significant differences are marked with asterisks (*p ≤ 0.05, ns, not statistically significant). Exact *p*-values are provided in the Source data file.

We further analyzed the efficacy of photosynthesis at different light intensities (Fig. 5; Supplementary Figs. 4–6) with a Dual-KLAS/NIR system, which allows for the deconvolution of redox changes in Fd, P700 and plastocyanin (PC), together with simultaneous chlorophyll fluorescence measurements^32^. Remarkably, at low intensities of photosynthetic light (13 µmol photons m^−2^ s^−1^), *pgrl1ab pgrl2-1* plants displayed a unique behavior with respect to thylakoid electron flow relative to plants expressing both PGRL1 and a functional PGR5 (Col-0 and *pgrl2-1*) or to plants without functional PGR5 (*pgrl1ab*, *pgr5-1*, *pgr5-1 pgrl2-1*) (Fig. 5a). More specifically, *pgrl1ab pgrl2-1* plants exhibited a significant decrease in the maximum quantum yield of PSII (Fv/Fm) (which went along with a markedly decreased Fm value, Supplementary Fig. 4) and in electron transport rates through PSII [ETR(II)] under low light compared to all other genotypes tested. In addition, regulated [Y(NPQ)] and nonregulated ([Y(NO)] energy dissipation in PSII were enhanced in *pgrl1ab pgrl2-1* (Fig. 5b). Conversely, the apparent PSI electron transport rate [ETR(I)] was increased and the PSI acceptor side was less limited [Y(NA)] in *pgrl1ab pgrl2-1* under low light (Fig. 5b). Although Fv/Fm levels in the *pgrl1ab pgrl2-1* lines were lower than those of the wild type, neither PSII complex assembly intermediates determined in BN/SDS-PAGE studies (Supplementary Fig. 7a) nor amounts of PSII marker subunits examined by immunodetection assays were affected (Supplementary Fig. 7b). Moreover, in *pgrl1ab pgrl2-1* plants the major light harvesting complex of PSII (LHCII) was preferentially dephosphorylated in darkness, similar to Col-0 and *pgrl2-1* plants (Supplementary Fig. 8).

At moderate light intensities (110 µmol photons m^−2^ s^−1^), equivalent to those used for propagation of the plants shown in Fig. 2, the effects of the mutations on photosynthesis were rather mild (Fig. 5a, Supplementary Fig. 5). However, some deviations from the WT control were detected for plants overexpressing *PGRL2* with respect to the parameters ETR(II), ETR(I), and Y(NA) (Fig. 5a, Supplementary Fig. 5). Notably, *pgrl1ab pgrl2-1* had the highest oxidized plastoquinone pool among all genotypes tested, as indicated by their relatively lowest 1-qL and 1-qP values (Supplementary Fig. 5d, e).

Under high light intensities (477 µmol photons m^−2^ s^−1^), thylakoid electron flow in *pgrl1ab pgrl2-1* plants was very similar to that of the other genotypes with a functional PGR5 (Col-0 and *pgrl2-1*)(Fig. 5a, Supplementary Fig. 6), implying that PSII and PSI could operate efficiently in the absence of PGRL1 and PGRL2. In fact, at this light intensity, two distinct clusters with clearly different photosynthetic effects could be discerned among the different genotypes (Fig. 5a). The *pgr5-1-*like cluster (*pgr5-1*, *pgrl1ab*, *pgr5-1 pgrl2-1*, and *P*_*35S*_*:PGRL2* #1 and #2) suffered from severe PSI acceptor-site limitation [Y(NA)] and consequently yielded lower ETR(II) and ETR(I) values, together with a substantially higher fraction of reduced Fd and conversely a lower fraction of oxidized PC compared to the WT cluster (Col-0, *pgrl2-1*, and *pgrl1ab pgrl2-1*) (Supplementary Fig. 6). Due to their low PMF (Fig. 3c), members of the *pgr5-1*-like cluster could not effectively build up NPQ (Supplementary Fig. 6f). Moreover, plants from the *pgr5-1-*like cluster with their more reduced plastoquinone pool (relative to WT) at high light (as determined by enhanced 1-qL and 1-qP values; Fig. 5a) displayed higher LHCII phosphorylation at 500 µmol photons m^−2^ s^−1^ compared to plants from the WT cluster including *pgrl1ab pgrl2-1* plants (Supplementary Fig. 8).

Taken together, these findings demonstrate that plants that accumulate PGR5 in the absence of PGRL1 and PGRL2 behave photosynthetically similar to plants with functional PGR5 and PGRL1 at moderate and high light intensities. But at very low light intensities they clearly deviate from the thylakoid electron flow observed in plants either lacking functional PGR5 or expressing both functional PGR5 and PGRL1. Indeed, the increased electron transport rate [Y(I)] and the less limited acceptor side [Y(NA)] of PSI (Fig. 5b) could be explained by increased CEF rates in *pgrl1ab pgrl2-1*, which were also inferred from analyses under light conditions with minimal photosynthetic electron transport rates (Fig. 4). As a consequence, a higher PMF could be built up leading to higher levels of non-photochemical quenching [Y(NPQ)] and lowered electron transport rates [ETR(II)] of PSII in *pgrl1ab pgrl2-1* (Fig. 5b). Thus, in the absence of both PGRL1 and PGRL2, PGR5 levels corresponding to only ~35% of wild-type levels (Fig. 3b) cause an unbalanced photosynthetic electron transport under low light.

### PGRL2 negatively regulates the stability of PGR5

**Fig. 6:**
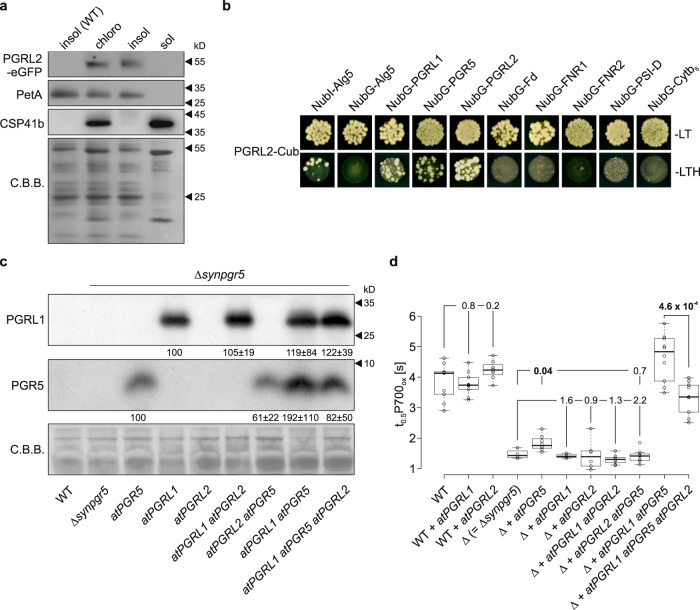
PGRL2 can interact with PGR5 and PGRL1, and destabilizes PGR5 in *Synechocystis*. **a** Chloroplast localization of PGRL2-eGFP. Chloroplasts were isolated from oePGRL2-eGFP plants and separated into insoluble (Insol) and soluble (Sol) fractions. The purity of the chloroplast fractions was assessed by immunodetection of PetA and CSP41b, which served as marker proteins for the insoluble and soluble chloroplast fractions, respectively. PVDF membranes stained with Coomassie brilliant blue (C.B.B.) served as loading controls. The experiment was repeated two times (technical replicates) with similar results. **b** Split-ubiquitin assays used to detect interactions between PGRL2 and CEF components. Assays were performed with fusions to the C-terminal (Cub) and N-terminal (NubG) halves of ubiquitin. NubI-Alg5 served as a positive control, Alg5 fused to NubG (NubG-Alg5) was the negative control. To test for interactions involving PGRL2, the mature PGRL2 protein (without its TP) was fused to Cub (PGRL2-Cub) and CEF components were fused to NubG. Yeast colonies were first plated on permissive medium (−LT, lacking Leu and Trp) and then on selective medium (−LTH, lacking Leu, Trp, and His). **c** Aliquots (40 µg) of total membrane proteins prepared from *Synechocystis* strains expressing PGRL2 in different genetic backgrounds were fractionated by SDS-PAGE and subjected to immunoblotting using PGR5- or PGRL1-specific antibodies. Representative blots from five experiments for atPGRL1 and nine experiments for atPGR5 are presented. Numbers below immunodetection signals correspond to average protein contents relative to atPGRL1-only and atPGR5-only expression strains ± standard deviation (for individual data points see Supplementary Fig. 10e). The PVDF membrane stained with Coomassie Brilliant Blue (C.B.B.) served as a loading control. Because amounts of PGRL2 were too low to be detected by immunoblotting, expression of its mRNA was monitored by Northern analysis (see Supplementary Fig. 10d). **d** Expression of PGRL2 in *Synechocystis* strains counteracts the effects of PGR5 on CEF. Values of t_0.5_P700_ox_ in PGRL2 expression strains, together with the appropriate controls are shown (*n* = 9/9/8/4/8/3/6/8/7/10/9, order as displayed). The horizontal lines represent the median, and boxes indicate the 25th and 75th percentiles. Whiskers extend 1.5× the interquartile range, outliers are represented as dots. Statistically significant differences according to Holm-corrected, two-sided Student’s t-tests are indicated in bold *p*-values (*p* ≤ 0.05). Brackets show groups tested for significant differences from the respective reference genotype (the leftmost in each group) and *p*-values are provided.

The mRNA expression profiles of *PGRL2* and *PGRL1* differ markedly during development and under diverse environmental conditions (Supplementary Fig. 9). The antibody we raised against PGRL2 detects the protein at concentrations of >0.01 mmol/[mol Chl], and since it fails to detect its target in WT plants (Supplementary Fig. 2d), PGRL2 must be at least 70-fold less abundant than the concentrations of PGRL1 previously found *in planta*^21^. Nevertheless, overexpressed PGRL2-eGFP was detectable in the insoluble fraction of chloroplast proteins (Fig. 6a), and PGRL2 accumulated in thylakoids to levels corresponding to around 25% of the amount of PGRL1 found in PGRL2 overexpressor lines (Supplementary Fig. 2d). In split-ubiquitin analyses, PGRL2 interacted only with itself, PGRL1 and PGR5, but (unlike PGRL1)^18^ not with other components of PSI or the cyt *b*_*6*_*f* complex (Fig. 6b).

Our data suggest that PGRL2 has a negative impact on PGR5 accumulation such that, in its absence, PGR5 levels rise—even in the absence of PGRL1, which itself might inhibit degradation of PGR5 by PGRL2. To test this hypothesis, we turned to our cyanobacterial testbed (Fig. 6c, d; Supplementary Fig. 10). We have recently shown that Arabidopsis PGR5 and PGRL1 function in the cyanobacterium *Synechocystis* and that the PGR5_G130S_ variant found in the Arabidopsis *pgr5-1* mutant fails to accumulate in *Synechocystis*^29^. Therefore, we tested whether the negative effect of PGRL2 on PGR5 accumulation can be recapitulated in *Synechocystis* by expressing PGRL2 in the presence of PGRL1 or PGR5, or both. This experiment was done in a strain that lacks the endogenous cyanobacterial PGR5 (synPGR5), in order to eliminate endogenous CEF activity (Supplementary Fig. 10). The effects of these different PGR5-PGRL1-PGRL2 combinations were monitored by immunoblot analyses and by quantifying the rate constant t_0.5_P700_ox_^29^ as a measure of PSI oxidation (Fig. 6c, d). In this system, we found that PGRL2 destabilizes PGR5, irrespective of whether PGRL1 is present or not (Fig. 6c, Supplementary Fig. 10). In consequence, t_0.5_P700ox values are also decreased when PGRL2 is co-expressed with either PGR5 alone, or in combination with PGRL1 (Fig. 6d). Taken together, these results clearly show that PGRL2 has a negative effect on PGR5 levels, and therefore on CEF activity.

### PGRL1 is not the target of antimycin A *in planta*

**Fig. 7:**
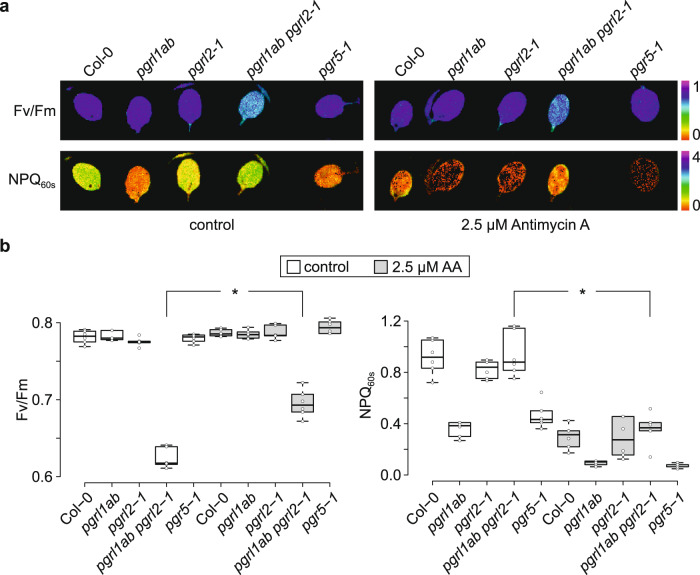
PGRL1 is not the target of antimycin A (AA) in planta. **a** The effect of AA on maximum quantum yield of PSII (Fv/Fm) and transient non-photochemical quenching (NPQ) was examined by Imaging PAM analyses. Leaves were infiltrated with 2.5 µM AA, dark-exposed for 5 min and subjected to Chl *a* fluorescence analysis. After an initial saturating blue light pulse for Fv/Fm determination, actinic blue light was switched on and further saturating light pulses were applied every 20 s. Transient NPQ values were recorded 60 s after light induction. Fv/Fm and NPQ of infiltrated, detached leaves are depicted on a false-color scale ranging from 0 to 1 and 0 to 4, respectively. **b** Boxplot analyses of Fv/Fm and NPQ values shown in panel a. Open circles represent data from six leaves, treated either with infiltration medium alone or supplemented with AA. The horizontal lines represent the median, and boxes indicate the 25th and 75th percentiles. Whiskers extend 1.5× the interquartile range, outliers are represented as dots. The effect of AA on *pgrl1ab pgrl2-1* was tested in a paired sample T-test (two-sided). Statistically significant differences are marked with asterisks (*p ≤ 0.05). The exact *p*-values are 4.6 × 10^−7^ and 3.0 × 10^−3^ in the left and right panel, respectively.

Previous experiments with PGR5 from *Pinus taeda* and on *A. thaliana* PGR5 proteins bearing amino-acid exchanges that mimic the sequence of *P. taeda* PGR5, which were performed either on ruptured chloroplasts or detached leaves, indicated that PGR5 might be the target of AA, which inhibits CEF^26^. Conversely, the ability of PGRL1 to reduce the quinone DMBQ in vitro is also inhibited by high concentrations of AA^21^. We re-evaluated the in vitro DMBQ assay and found it to be rather unspecific, because L-cysteine alone already reduces the absorption at 260 nm, and thus mimics DMBQ reduction (Supplementary Fig. 11). Indeed, this reaction (‘thiol addition’) is known to occur between other quinones and L-cysteine^33–35^. We then infiltrated leaves from different genotypes with AA and determined Fv/Fm and transient NPQ values after 60 s of illumination as a measure for CEF, in order to study whether CEF can also be inhibited by AA in *pgrl1ab pgrl2-1* plants, which contain PGR5, but lack PGRL1 and PGRL2 (Fig. 7a, b). Interestingly, AA infiltration led to partial restoration of the Fv/Fm phenotype detected for *pgrl1ab pgrl2-1* (Fig. 5b), whereas PSII functionality of the other genotypes was not substantially impaired by AA treatment (Fig. 7b, Supplementary Fig. 12). As expected from the results described above (see Fig. 3b), in control experiments without AA treatment all genotypes in which PGR5 accumulates (WT, *pgrl2-1* and *pgrl1ab pgrl2-1*) displayed higher transient NPQ values than *pgr5-1* and *pgrl1ab* plants devoid of the protein. In contrast, AA treatment suppressed NPQ induction in all genotypes including *pgrl1ab pgrl2-1*, implying that AA acts on PGR5 to inhibit CEF. A minor drop in NPQ induction after AA treatment was observed in the two genotypes without PGR5 (*pgr5-1* and *pgrl1ab*), but this can be attributed to indirect effects on other AA targets, such as cytochrome b_559_ in PSII or respiratory electron transport in mitochondria^36,37^.

In summary, our results indicate that AA inhibits CEF independently of PGRL1 and PGRL2.

## Discussion

Our results clearly show that PGRL1 is not the FQR in CEF, a hypothesis^21^ that was primarily based on an in vitro DMBQ assay which, as we show here, is not specific enough to measure FQR activity (see Supplementary Fig. 11). In fact, both PGR5 accumulation and CEF occur in the absence of both PGRL1 and PGRL2 (see Fig. 3), which prompts two major questions. Why do plants use three proteins (PGR5, PGRL1, and PGRL2) for CEF, if one of them (PGR5) is sufficient for the task? And what makes PGRL1 and PGRL2 so vital that they are present together in all plants and many green algae (Supplementary Fig. 1)?

**Fig. 8:**
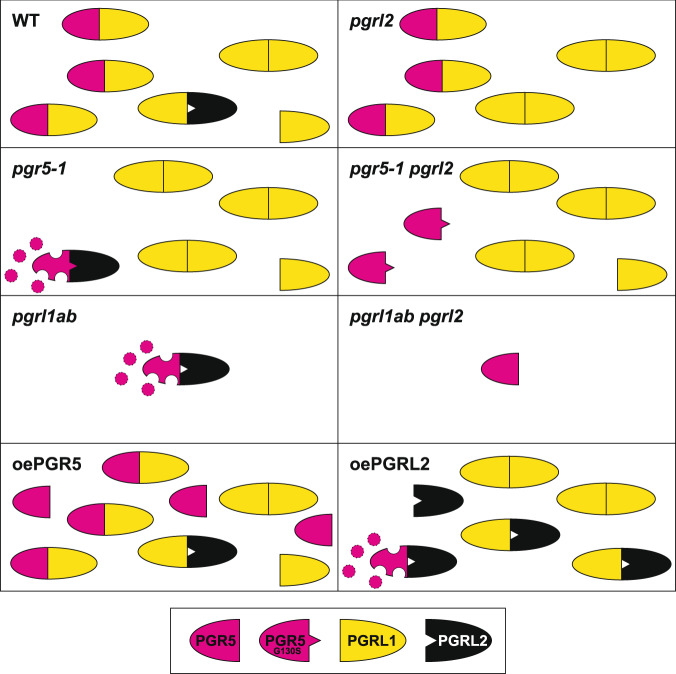
Model for PGRL1-PGRL2-dependent regulation of PGR5 accumulation. This model is based on the following observations and assumptions (see main text). (i) The ratios of PGRL1 to PGR5 to PGRL2 in WT cells are approximately 70:10:<1. (ii) PGRL1 and PGR5 form heterodimers to stabilize PGR5, safeguard its activity and prevent its PGRL2-dependent degradation. (iii) PGRL2 can interact with PGRL1 and PGR5, and its interaction with PGR5 triggers degradation of PGR5 (symbolized by disintegration of the symbol for PGR5). (iv) PGRL1 cannot interact with and stabilize the mutated PGR5 (PGR5_G130S_), which allows it to interact with PGRL2 and be degraded. WT plants accumulate all of the three proteins, and PGR5 is rendered inaccessible to PGRL2 owing to the formation of either PGR5-PGRL1 and/or PGRL2-PGRL1 heterodimers (in the Figure both possibilities are depicted). Loss of PGRL1 (in *pgrl1ab* plants) destabilizes PGR5, because PGR5 becomes accessible to PGRL2. Loss of PGRL2 (in *pgrl2-1* plants) has no obvious effect when PGRL1 and PGR5 are present, but precludes degradation of PGR5 if PGRL1 is also absent (as in *pgrl1ab pgrl2-1*). Mutated PGR5 (PGR5_G130S_) can accumulate when PGRL2 is absent (in *pgr5-1 pgrl2-1*) but is not functional (see main text). Its degradation in *pgr5-1* plants might be related to its inability to interact with PGRL1 (indicated by the spike in the symbol for PGR5_G130S_ that prevents it from interacting with PGRL1, but allows it to engage with PGRL2). Overexpression of PGR5 (oePGR5) or PGRL2 (oePGRL2) leads to accumulation of harmful “free” PGR5 and degradation of PGR5, respectively.

The answer to the first question is obvious. Although “free” PGR5 suffices to allow and even increase CEF in the absence of PGRL1 (see Figs. 3 and 4), it appears to impair thylakoid electron flow in low light (see Fig. 5) and plant growth under CL conditions (Fig. 2). Indeed, both WT plants and mutants lacking PGRL1 and/or PGR5 (*pgr5-1* and *pgrl1ab*) grow better under CL conditions and display patterns of thylakoid electron flow in low light that markedly differ from those of plants with “free” PGR5 (*pgrl1ab pgrl2-1*) (see Figs. 2 and 5). This implies that “free” PGR5 is more detrimental to plants than having no PGR5 at all, at least under certain light conditions. In line with this, we propose that also the detrimental effects of overexpression of PGR5 on plant and cyanobacterial growth^27,28,38^ are attributable to “free” PGR5 (see Fig. 8). In fact, the CEF activity of *pgrl1ab pgrl2-1* plants, as determined by chlorophyll fluorescence and P700 oxidation analysis (see Fig. 4), is similar to the one observed for PGR5 overexpressors in *A. thaliana*^27^, suggesting that the primary reason for these impairments is the activity of “free” PGR5 which is normally masked by PGRL1. Moreover, this feature of PGR5 might also contribute to its destabilization, because the level of the mutated PGR5 protein in *pgr5-1 pgrl2-1* plants is about twice as high as the amount of WT PGR5 found in *pgrl1ab pgrl2-1* plants (Fig. 3a). It has been suggested that ferredoxin may reduce a low potential variant of Q_A_ in a subpopulation of PSII^39^, but increased PSII photoinhibition or damage due to enhanced transport of electrons to PSII induced by “free” PGR5 appears to be rather unlikely, since PSII assembly and accumulation of representative PSII subunits (see Supplementary Fig. 7) was WT-like in *pgrl1ab pgrl2-1*.

What functions are served by PGRL1 and PGRL2? For PGRL2 the answer is clear: it triggers the degradation of PGR5, but its action only becomes evident under conditions in which either PGRL1 is absent (in *pgrl1ab*), PGR5 is mutated (in *pgr5-1*) or PGRL2 is present in relative excess (in the *Synechocystis* system and in the PGRL2 overexpressors *in planta*; Figs. 3 and 6). We, therefore, conclude that PGRL2 can only trigger PGR5 degradation *in planta* when PGR5 is unable to productively interact with PGRL1. For the mutated PGR5 found in the *pgr5-1* strain (PGR5_G130S_), a perturbation in its interaction with PGRL1 has been indirectly shown in the heterologous *Synechocystis* system^29^, where PGRL1 stabilizes WT PGR5 (i.e., increases its accumulation relative to PGR5 expressed alone), but not PGR5_G130S_. PGR5_G130S_ accumulates to about 70% of WT PGR5 levels in plants devoid of PGRL2, and PGRL1 levels are reduced in *pgr5-1 pgrl2-1* and are even lower in *pgr5-1*. Both observations could in principle be explained by assuming that formation of PGR5-PGRL1 complexes stabilizes both proteins. However, in the *Synechocystis* system, WT PGR5 fails to stabilize PGRL1^29^. Moreover, we could not detect yet any marked physiological effect of absence of PGRL2 alone (in *pgrl2-1* plants) such that the physiological function of PGRL2 remains elusive and the molecular mechanism of how PGRL2 can remove PGR5 from PGRL1-PGR5 complexes and trigger its degradation remain to be elucidated.

What is the molecular function of PGRL1? Obviously, it protects PGR5 against PGRL2-dependent destruction, and this might explain why PGRL1 is much more abundant than PGRL2 (ref. ^21^ and Supplementary Fig. 2). In fact, PGRL1 might protect PGR5 against PGRL2, either by forming heterodimers with PGR5 and preventing PGRL2 from doing so, or by directly interacting with PGRL2, thus sequestering it from PGR5 (see Fig. 8). Actually, our split-ubiquitin results reported earlier^18^ and in this study (see Fig. 6b) are compatible with both possibilities. But the PGRL1-PGR5 interaction appears to serve additional purposes beyond protecting PGR5 from the action of PGRL2, because PGRL1 stabilizes PGR5 in terms of increasing its abundance (see above) and it seems to modulate (or channel) PGR5 activity.

From the evolutionary viewpoint, PGRL2 appears in the green lineage together with PGRL1, but is not found in the green alga *Chlamydomonas reinhardtii*^30^. In fact, *C. reinhardtii* and *A. thaliana* differ markedly with respect to CEF. Thus, in *C. reinhardtii*, CEF employs a PSI-cyt *b*_*6*_*f* supercomplex that is insensitive to AA^12^ (although AA sensitivity appears to vary among different *C. reinhardtii* strains^40^), and recently a second CEF pathway involving an alternative Fd-assisted Q cycle of the cyt *b*_6_*f* complex was proposed^41^. Moreover, inactivation of PGRL1 alone (in the presence of functional PGRL2) prevents PGR5 accumulation and CEF in *A. thaliana*^18^, whereas in *C. reinhardtii* in the absence of PGRL1 small amounts of PGR5 can accumulate^42^ and CEF still occurs^42,43^. In line with this, downregulation of ANR1 or CAS, two central components of the *C. reinhardtii* PSI- cyt *b*_*6*_*f* supercomplex, has much more drastic effects on CEF than lack of PGR5 or PGRL1 alone^44^, and CEF supercomplex formation was observed in *Chlamydomonas pgr5 pgrl1* strains^13^. This strongly suggests that at least part of the CEF mechanism related to the supercomplex in *Chlamydomonas* does not require PGRL1 and PGR5. With respect to PGRL2 this allows to conclude that *C. reinhardtii* might not require PGRL2 to control PGR5 accumulation, because PGR5 might already be safely embedded in the supercomplex^45^ and/or be unable to compromise thylakoid electron flow if present in its “free” form. More generally, it is worthwhile to note that at least some of the controversial discussions on the function of PGR5 and PGRL1 can be resolved when it is considered that results from *C. reinhardtii* and *A. thaliana* are not directly comparable in the light of the aforementioned fundamental differences in the structure and mechanism of CEF in the two organisms. In consequence, future studies will have to clarify whether and to which extent the molecular function of PGR5 is comparable in *A. thaliana* and *C. reinhardtii*. For instance, mutation of the only cysteine residue in PGR5 has no apparent effects on CEF in *C. reinhardtii*^41^ and it needs to be tested whether this also holds true for CEF activity in *A. thaliana*.

In summary, PGR5 emerges as the central player in CEF. In its “free” form it appears to have harmful side-effects and is unstable, and therefore requires PGRL1 and PGRL2 for its primary function.

## Methods

### Plant material and growth conditions

*Arabidopsis thaliana* plants, wild-type and mutant, were grown on soil under control light (CL, photoperiod of 12 h light/12 h darkness with a light intensity of 100 µmol photons m^−2^ s^−1^ in the light phase) or under fluctuating light (FL) conditions (i.e., cycles of 5 min at 50 μmol photons m^−2^ s−^1^ and 1 min at 500 μmol photons m^−2^ s^−1^) during the day. In all cases, temperatures (22 °C/20 °C during the day/night cycle) and relative humidity (60%) were strictly controlled. Fertilizer was added according to the manufacturer’s recommendations (Osmocote Plus; Scotts Deutschland).

The Arabidopsis *pgr5-1* and *pgrl1ab* mutants have been described previously^17,18^ and the *pgrl2-1* T-DNA line (SALK_037265C) was obtained from the SALK collection^46^. Double and triple mutants (*pgr5-1 pgrl2-1*, and *pgrl1ab pgrl2-1*) were generated by crossing the respective single- and double-mutant parental lines. Genomic DNA was extracted as described^47^ and F_2_ plants were screened by PCR using gene- and T-DNA-specific primer combinations (see Supplementary Table 2). The *pgr5-1* allele was analyzed by amplifying and sequencing the genomic region spanning the S130 point mutation in *AT2G05620*^17^.

For *PGRL2* overexpression, the *AT5G59400* coding region (gene model *AT5G59400.1* according to TAIR) was cloned into the binary Gateway destination vector pH2GW7^48^, placing the coding sequence under the control of the 35S promoter.

Plasmids were transformed into *Agrobacterium tumefaciens* cells (GV3101), which were then employed for plant transformation^49^. Seeds from *P*_*35S*_:*PGRL2* Col-0 transformations were sterilized by treatment with chlorine gas for 4 h, and positive transformants were selected on Murashige and Skoog salt medium (1×) containing 25 µg mL^−1^ hygromycin, 0.8% [w/v] plant agar, 1% [w/v] sucrose and 200 µg mL^−1^ cefotaxim. Overexpression of *PGRL2* was monitored by Western analysis and two independent lines were propagated for further experiments.

### Complementation of the *pgrl1ab pgrl2-1* mutant phenotype

To rescue the *pgrl1ab* mutant phenotype, the genomic DNA region of *AT5G59400* was amplified with primers binding 220 bp up- and 242 bp downstream of the start and stop codons, respectively (see Supplementary Table 2 for sequence information). The fragment was then cloned into the binary Gateway destination vector pHGW^48^ using the Gateway cloning system (Invitrogen, Carlsbad, CA, USA) according to the manufacturer’s instructions. After transformation into *A. tumefaciens* (GV3101), *pgrl1ab pgrl2-1* plants were subjected to the floral-dip transformation procedure as described^49^. Stable transformants were selected as described in the foregoing paragraph, transferred onto soil and kept for four weeks under control light conditions. Complementation was verified by Dual-KLAS/NIR measurements, and two independent lines showing a *pgrl1ab*-like transient NPQ were further analyzed by Northern and Western blotting.

### Chl *a* fluorescence, P700, and ECS measurements

In vivo chlorophyll *a* fluorescence, P700 absorbance changes, as well as plastocyanin (PC) and ferredoxin (Fd) redox states were simultaneously monitored on single attached leaves using a Dual/KLAS-NIR spectrophotometer (Walz, Effeltrich, Germany). Absorbance ratios at different wavelengths (785/840 nm, 810/870 nm, 870/970 nm and 795/970 nm) were analyzed and changes in redox states of P700, PC and Fd were deconvoluted based on differential model plots for P700, PC, and Fd^32,50^. Saturating pulses of white light (8000 μmol photons m^−2^ s^−1^ for 0.3 s) were applied to determine photosynthetic parameters, which were calculated by the DUAL/KLAS-NIR software based on the equations described previously^32,51–53^. Light induction and dark recovery curves were constructed for attached leaves, which had been dark-adapted for 30 min. Curves were plotted using blue actinic light with a light intensity of either 13, 110, or 477 μmol photons m^−2^ s^−1^ for 6 min, followed by 3 min of darkness. Saturating pulses were applied every 20 s. CEF induction was examined by analyzing the transient rise in non-photochemical quenching (NPQ) at 110 μmol photons m^−2^ s^−1^ during dark-light induction experiments^17,18^.

Steady-state ECS signals were monitored on single attached leaves using the Dual-PAM-100 (Walz, Effeltrich, Germany) equipped with a P515/535 emitter-detector module (Walz, Effeltrich, Germany) as described before^54^. Plants were dark-adapted for 30 min and subjected to a single turnover flash of the P515 absorbance change signal (ECS_st_). After exposure to actinic light (340 µmol photons m^−1^ s^−1^) for 15 min, the light was turned off and dark-interval relaxation kinetics were recorded. The difference in PMF across the thylakoid membrane (ECS_T_) was estimated from the total amplitude of the rapid P515 signal decay after transition to darkness^55^ and normalized to ECS_st_.

Estimation of the plastoquinone reduction state and P700 oxidation kinetics were analyzed simultaneously as described^27^ with the Dual-PAM-100 system (Walz, Effeltrich, Germany). Plants were dark-adapted 1 h before and attached leaves were exposed to measuring light intensity of 1 μmol photons m^−2^ s^−1^ which corresponded to a ~10-fold higher intensity compared to standard PAM fluorometry analyses. After 60 s, far-red light (FR) was switched on for 1 min to preferentially excite PSI. During the 3 min experiment, a saturation pulse (SP) was given at the time of 30 s and 90 s, respectively. The degree of plastoquinone reduction was estimated from the difference between the minimal Chl *a* fluorescence level shortly before (Fo’) and during FR light exposure (Fo_FR_) which was referred to the maximal Chl *a* fluorescence yield Fm according to the formula (Fo’−Fo_FR_)/Fm (see Fig. 4a). P700 oxidation kinetics were recorded by following the difference of the 875 and 830 nm transmittance signals and the oxidation half time of P700^+^ denoted as t_0.5_P700_ox_ was determined from the P700 signal rise after FR light exposure.

### Antimycin A treatments

Infiltration of antimycin A (AA) into intact leaves was carried out as described^26^. Several leaves from different genotypes were vacuum-infiltrated with infiltration medium (300 mM sorbitol, 5 mM MgCl_2_, 2.5 mM EDTA, 20 mM HEPES/KOH pH 7.6) optionally supplemented with 2.5 µM AA. Leaves were dark-exposed for 5 min before being subjected to Imaging-PAM analyses (Walz, Effeltrich, Germany). After Fv/Fm determination of dark-adapted leaves, actinic blue light was switched on (100 µmol photons m^−2^ s^−1^) and NPQ values were recorded by applying saturating light pulses every 20 s.

### Northern analysis

Total leaf RNA was isolated from four-week-old plants (Col-0, *pgrl2-1*, *pgrl1ab pgrl2-1*, and two *gDNA-PGRL2 pgrl1ab pgrl2-1* lines) using the TRIzol reagent (Invitrogen). Northern analyses were carried out as described^56^. In brief, 25 µg of total RNA was size-fractionated on formaldehyde-containing agarose gels (1.5% [w/v]) and transferred onto nylon membranes (Hybond-N+, Amersham Bioscience). After crosslinking by irradiation with UV light (Stratalinker UV Crosslinker 1800), equal loading was checked by staining nylon membranes with methylene blue dye (0.02% [w/v] methylene blue, 0.3 M sodium acetate, pH 5.5). *PGRL2*-specific transcripts were detected by employing probes that bind either up- (probe 1) or downstream (probe 2) of the T-DNA insertion site in *pgrl2-1* (see Supplementary Table 2 for sequence information). To this end, PCR products were amplified from cDNA, which was reverse-transcribed from Col-0 RNA (SuperScript III reverse transcriptase; Invitrogen), labeled with radioactive [α-^32^P]dCTP and used for hybridization under stringent conditions^57^. After several washing steps, signals on nylon membranes were detected with the Typhoon Phosphor Imager System (GE Healthcare, Chicago, IL, USA).

### Suborganellar localization of PGRL2-eGFP

The coding sequence of *PGRL2* without its stop codon (see also Supplementary Table 2) was cloned into the binary Gateway vector pB7FWG2.0^48^ and introduced into the *pgrl2-1* background as described above. Stable transformants overexpressing *PGRL2-eGFP* were selected by exposure to several rounds of BASTA treatment, followed by immunodetection of PGRL2-eGFP using PGRL2-specific antibodies generated in this study. Chloroplasts were isolated from the next generation as described^58^ and were further separated into soluble and insoluble fractions as described^56^. In brief, leaf material from 5-week-old, dark-adapted plants was homogenized in buffer containing 330 mM Sorbitol, 20 mM Tricine/NaOH (pH 7.6), 5 mM EGTA, 5 mM EDTA, 10 mM NaHCO_3_, 0.1% (w/v) BSA, and centrifuged for 5 min at 1500 × *g* (4 °C). Crude chloroplasts were resuspended in 300 mM Sorbitol, 20 mM HEPES/KOH (pH 7.6), 5 mM MgCl_2_, 2.5 mM EDTA, applied to a two-step Percoll gradient (40–80% (v/v)), and centrifuged at 6500 × *g* for 20 min at 4 °C. Intact chloroplasts were collected from the interface and ruptured in 20 mM HEPES/KOH (pH 7.5), 10 mM EDTA for 30 min on ice. Stromal proteins were separated from the membranous fraction by centrifugation (42,000 × *g*, 30 min, 4 °C). Each fraction was assessed for purity and the localization of PGRL2-eGFP fusion protein (detected by a PGRL2 antibody dilution of 1/2,000) was determined using appropriate immunodetection assays [CSP41b^59^ (detected by a CSP41b antibody dilution of 1/5000, provided by David Stern) and PetA (detected by a PetA antibody dilution of 1/5000, Agrisera, Vännäs, Sweden) served as markers for the soluble and insoluble fractions, respectively].

### Split-ubiquitin assay

Transient interactions of PGRL2 with putative components of cyclic electron transport were probed with split-ubiquitin assays using the Dual Membrane kit (Dualsystems Biotech AG). The coding sequence corresponding to PGRL2_41-299_ (without the transit peptide) was cloned into the vectors pAMBV4 and pADSL-Nx (Dualsystems Biotech AG) according to the supplier’s instructions (see also Supplementary Table 2). To test whether PGRL1A interacts with PGRL2, the coding sequence corresponding to PGRL1A_61-324_ (without the transit peptide) was cloned into pADSL-Nx. PGRL2-Cub interaction assays in the DSY-1 yeast strain (Clontech, Palo Alto, CA) co-transformed with NubG-PGRL1, NubG-PGR5, NubG-PGRL2, NubG-FNR1, NubG-FNR2, NubG-PSI-D, and NubG-Cytb_6_ constructs were carried out as described^18,60^. All NubG constructs were checked for auto-activation in the yeast background carrying the construct coding for Alg5-Cub. No auto-activation was detected.

### Bioinformatic analysis

Transit peptide sequences and transmembrane domains were predicted by ChloroP (http://www.cbs.dtu.dk/services/ChloroP/) and TMHMM (http://www.cbs.dtu.dk/services/TMHMM/), respectively.

Gene-expression analyses of *PGRL2* (*AT5G59400*) and *PGRL1A* (*AT4G22890*) were conducted with Genevisible (https://genevisible.com/search). The alignment in Fig. 1 was built with Vector NTI and formatted with Boxshade (https://embnet.vital-it.ch/software/BOX_form.html). The phylogenetic tree was constructed with the CLC workbench software (v8.1).

Hierarchically clustered heat maps of photosynthetic parameters measured in light-saturation-curve analyses were generated by ClustVis^61^.

### Immunoblot analyses and PGRL2 antibody generation

Rosette leaves (50 mg fresh weight) were ground in liquid nitrogen and homogenized in 500 µL of 2× Tricine buffer containing 8% [w/v] SDS, 24% [w/v] glycerol, 15 mM DTT and 100 mM Tris/HCl pH 6.8. The homogenate was incubated for 5 min at 70 °C and centrifuged for 10 min at 13,000 × *g*. Solubilized leaf proteins corresponding to 1 mg (for PGRL1 detection) and 3 mg (for PGR5 detection) fresh weight were loaded onto Tricine-SDS-PAGE gels^62^. Resolved proteins were transferred to polyvinylidene fluoride (PVDF) membranes (Immobilon-P; Millipore, Burlington, MA, USA) as described^29^. Equal loading was verified by staining PVDF membranes with Coomassie blue G-250 dye as described^62^. After blocking with TBS-T (10 mM Tris, pH 8.0, 150 mM NaCl, and 0.1% Tween 20) supplemented with 3% [w/v] BSA, PVDF membranes were probed with antibodies against PGR5 (1/2500 dilution; provided by Prof. T. Shikanai) and PGRL1 (1/10,000)^18^. Signals were visualized with enhanced chemiluminescence using the Pierce™ ECL western blotting substrate reagent (Thermo Fisher Scientific, Waltham, MA, USA) and an ECL reader system (Fusion FX7; VWR, Radnor, PA, USA). Signals were quantified with Bio-1D (version 15.03, Vilber Lourmat, Eberhardzell, Germany).

Antibodies against the N-terminal sequence of PGRL2 were raised in rabbits. To this end, the coding sequence for PGRL2_41-137_ was cloned into the expression vector pMal-c5x (NEB, Ipswich, MA, USA), resulting in the fusion of the maltose-binding protein (MBP) to PGRL2_41-137_. After transformation into BL21 (DE3) *Escherichia coli* cells (Thermo Fisher Scientific), heterologous expression and purification of MBP-PGRL2_41-137_ by affinity chromatography on amylose resin were carried out according to the manufacturer’s instructions. Purified MBP-PGRL2_41-137_ was then employed for commercial antibody production in rabbits (Pineda, Berlin, Germany). The final antiserum was subjected to affinity purification on immobilized MBP-PGRL2_41-137_. Dilutions of 1/2,000 were used for immunodetection assays of PGRL2.

### Quantification of PGRL2 overexpression

PGRL2 amounts in thylakoid membranes isolated from two independent *P*_*35S*_:*PGRL2* Col-0 and *P*_*35S*_:*PGRL2 pgrl1ab* transformants were quantified by immunodetection assays and compared to signals from known, titrated amounts of purified 6xHis-PGRL2_41-137_. Briefly, the coding sequence corresponding to PGRL2_41-137_ was cloned into pET151 (Invitrogen) as described in the supplier’s instructions (see also Supplementary Table 2 for sequence information). After transformation into BL21 (DE3) *Escherichia coli* cells (Thermo Fisher Scientific) and heterologous expression, purification of 6×His-PGRL2_41-137_ was carried out by making use of nickel nitrilotriacetic acid (Ni-NTA) agarose beads (Protino®, Macherey-Nagel, Düren, Germany). The amounts of 6xHis-PGRL2_41-137_ in elution fractions were quantified with the Bio-Rad protein assay (Bio-Rad, Hercules, CA, USA). Thylakoid membranes were isolated from 5-week old *P*_*35S*_:*PGRL2* Col-0 and *P*_*35S*_:*PGRL2 pgrl1ab* plants as described^63^. Chlorophyll concentration was determined as described^64^. Thylakoid samples (in Supplementary Fig. 2, 100% corresponds to 2.5 μg Chl or 2.78 nmol Chl) were fractionated together with titrated amounts of purified 6×His-PGRL2_41-137_ (1.1, 0.4, 0.2, and 0.1 pmol) by Tricine-SDS-PAGE^62^. Western analyses and immunodetection were performed as described above. PGRL2-specific signals were quantified with the Bio-1D software (version 15.03, Vilber Lourmat, Eberhardzell, Germany) and PGRL2 amounts were calculated in mmol/[mol Chl].

### *Synechocystis* mutant generation

*Synechocystis* strains expressing PGR5 and/or PGRL1A were generated as already described^29^. PGRL2 expression strains were generated by transformation with the genomic insertion vector pP2. Mature PGRL2 (lacking aa 1–41) was expressed under the control of the *Synechocystis psbA2* promoter from the *slr0319* locus (encoding β-lactamase blaOXA-3). For all expression strains successful transformation and segregation was confirmed by PCR.

### *Synechocystis* Northern blot, immunoblot and P700 oxidation state analyses

Northern blot analysis of *PGRL2* transcripts in *Synechocystis* was performed as described^29^ on 12.5-µg aliquots of total cellular RNA per strain. Radioactive probes were identical to those used for *Arabidopsis*.

Western blot analyses and P700 PAM measurements were performed as described^29^.

### Statistical analyses

Boxplots were created using BoxPlotR^65^. The horizontal lines represent the median and boxes indicate the 25th and 75th percentiles. Whiskers extend 1.5× the interquartile range, outliers are represented as dots. Statistical analyses were carried out in *R* v3.5.2 (https://www.r-project.org/). First, data were subjected to Shapiro-Wilk tests to check whether they were normally distributed. In case of deviations from normality, non-parametric tests were conducted and Kruskal–Wallis tests followed by pairwise Dunn’s tests were performed using the *R* package *dunn.test*. The *p*-values were adjusted on an experiment level using the Benjamini–Hochberg method. Statistically significant differences are indicated with asterisks (*p ≤ 0.05, ns, not statistically significant).

In the case of comparisons of *t*_0.5_ values for *Synechocystis* P700 oxidation rates, statistically significant differences were tested for by one-way ANOVA, followed by Bonferroni-Holm correction for multiple testing. ANOVA and Bonferroni-Holm correction were performed using the One-way ANOVA with post-hoc Test tool as implemented by Navendu Vasavada (https://astatsa.com/).

To examine the effect of AA treatment on Fv/Fm and NPQ_60s_ parameters for *pgrl1ab pgrl2-1* lines, a paired sample *T*-test (two-sided) was carried out using the *R* v3.5.2 package *t.test*.

### Accession numbers

ATG accession numbers: PGR5 (At2g05620), PGRL1A (At4g22890), PGRL1B (At4g11960), PGRL2 (At5g59400).

### Reporting summary

Further information on research design is available in the Nature Research Reporting Summary linked to this article.

## Supplementary information

Supplementary Information

Reporting Summary

## Data Availability

The authors declare that all data presented in this study are available in the figures and the accompanying Supplementary Information file. Data that support the study are available from the corresponding author upon reasonable request. Source data are provided with this paper.

## Source data

Source Data
