# Supplementary material for: PGRL2 triggers degradation of PGR5 in the absence of PGRL1
Source: Nat Commun. 2021 Jun 24;12:3941. doi: 10.1038/s41467-021-24107-7 (PMC8225790; doi:10.1038/s41467-021-24107-7)
Supplement: Supplementary file 1 — Supplementary Information [file 41467_2021_24107_MOESM1_ESM.pdf]

Supplementary Information

PGRL2 triggers degradation of PGR5 in the absence of PGRL1

Thilo Rühle, Marcel Dann, Bennet Reiter, Danja Schünemann, Belen Naranjo,
Jan-Ferdinand Penzler, Tatjana Kleine & Dario Leister

Nature Communications, 2021

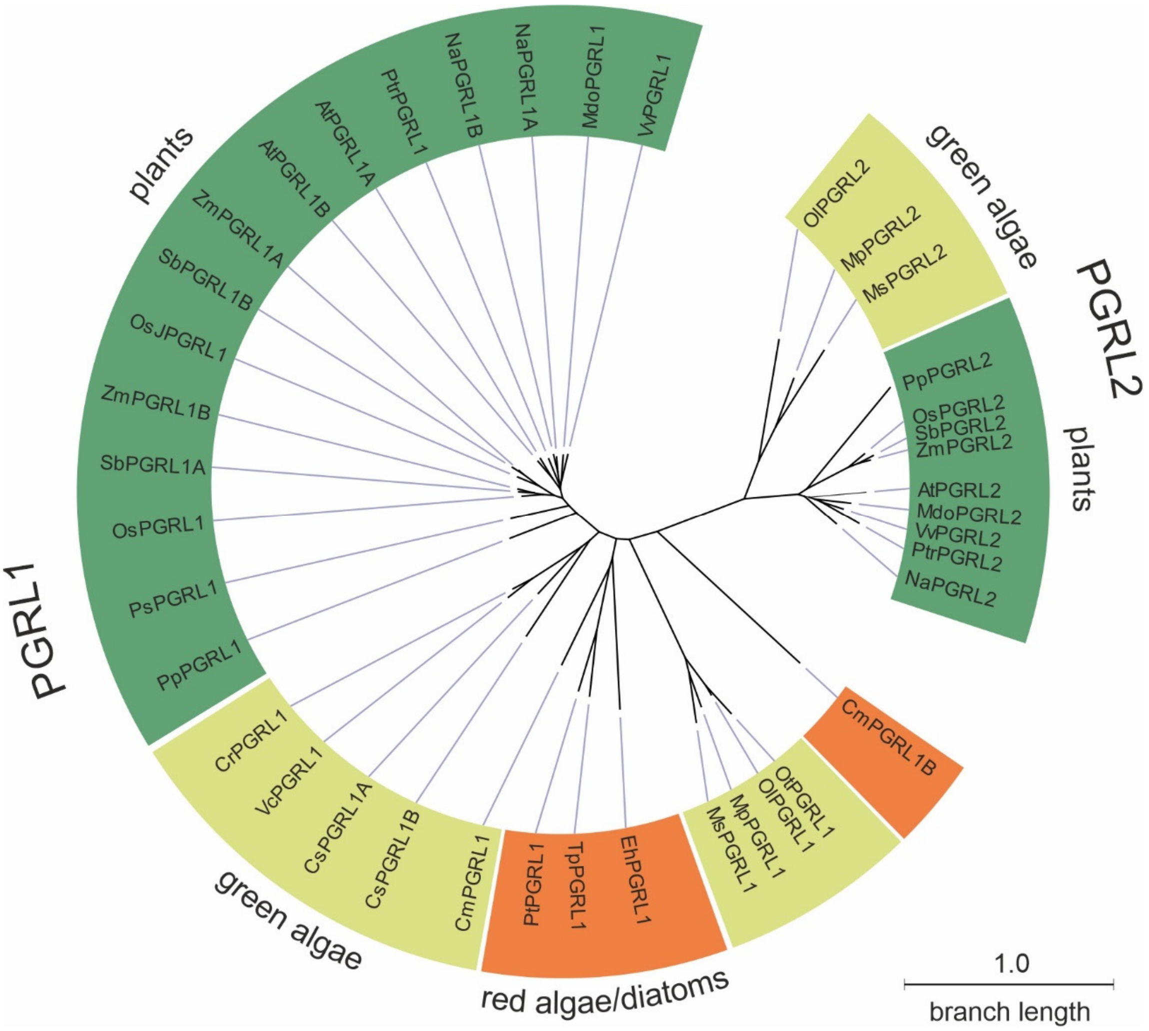

Supplementary Figure 1 Molecular phylogenetic analysis of the evolutionary relationships between PGRL1 and PGRL2 proteins in algae and plants.

The evolutionary history of the two proteins was inferred from sequence comparisons using the maximum-likelihood method. The analysis involves 40 amino-acid sequences (sequence identifiers are explained in **Supplementary Table 1**). A bootstrap analysis with 1000 replicates was carried out. Bold lines indicate a bootstrap value >50%. Alignment and evolutionary analyses were conducted with the CLC Workbench Software (Version 8.1). PGRL1 and PGRL2 sequences of plants and green algae are highlighted in dark and light green, respectively. PGRL1 sequences of red algae and diatoms are shown in orange. The scale bar indicates branch length in terms of amino-acid substitutions per site.

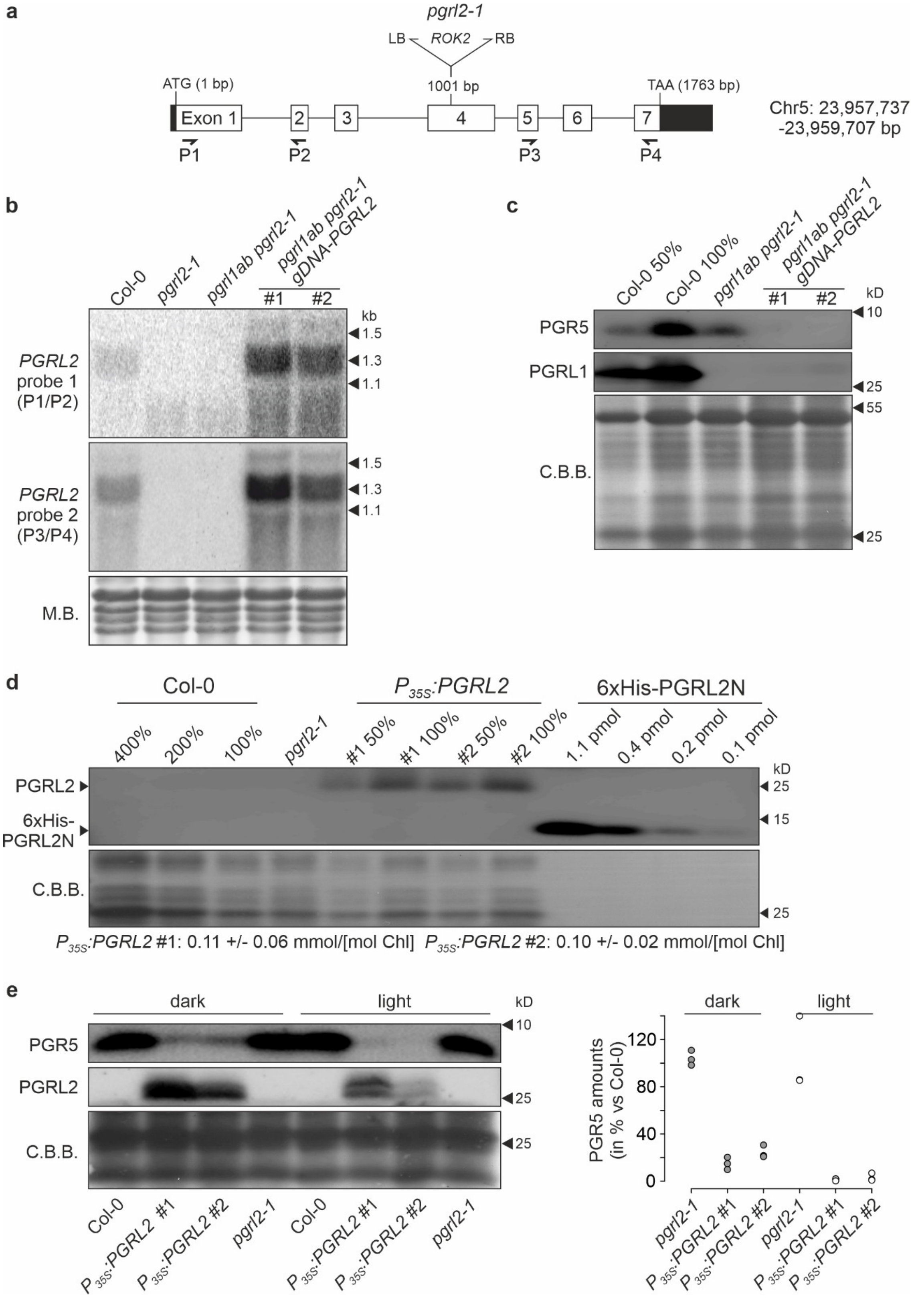

Supplementary Figure 2 Characterization of PGRL2: its knock-out mutant, complementation, overexpression and quantification of protein abundance.
a Structure of the *AT5G59400* gene and location of the T-DNA insertion in *pgrl2-1*. Left (LB) and right (RB) T-DNA borders are indicated. Exons are numbered and shown as white rectangles, UTRs as black rectangles. **b** Detection of *PGRL2* (*AT5G59400*) transcripts by Northern analyses. Two different probe pairs were used that bind either upstream (primer pair

P1/P2, shown in panel **a**) or downstream (primer pair P3/P4, shown in panel **a**) of the T-DNA insertion site in *pgrl2-1*. Nitrocellulose membranes were stained with methylene blue (M.B.) to provide loading controls. **c** Molecular analysis of the suppression of the *pgrl1ab* phenotype by *pgrl2-1* (and its complementation by a genomic fragment containing the *PGRL2* gene) using PGR5 and PGRL1 immunodetection assays. Note that functional complementation of the two lines was demonstrated by growth tests under fluctuating light conditions, tNPQmax and ECS_T/ECS_{st} measurements (see **Supplementary Fig. 3**). **d** Verification of PGRL2 overexpression. Dilutions containing known amounts of purified 6xHis-PGRL2N were size-fractionated by SDS-PAGE, together with thylakoids isolated from Col-0, *pgrl2-1* and P_{35S}*:PGRL2* (#1 and #2) plants (100% Col-0 corresponds to 2.5 µg Chl). Signals were immunodetected with a PGRL2N-specific antibody and quantified using Bio-1D software (Vilber Lourmat, Eberhardzell, Germany). Amounts of PGRL2 are expressed in mmol/[mol Chl]. Since WT levels are below the detection threshold of 0.01 mmol/[mol Chl], the PGRL1/PGRL2 ratio in WT plants is greater than 70. In effect, PGRL2 (< 0.01 mmol/[mol Chl]) is much less abundant than either PGR5 (0.09 mmol/[mol Chl]) or PGRL1 (0.7 mmol/[mol Chl]). Ratios of PGRL2 to PGRL1 in P_{35S}*:PGRL2* plants are 0.27 +/- 0.16 (line #1) and 0.24 +/- 0.06 (line #2), assuming that only ~60% of WT levels of PGRL1 is present in P_{35S}*:PGRL2* plants (Fig 3a) **e** Light-dependent degradation of PGR5 in Col-0 P_{35S}*:PGRL2* lines. Leaves were collected from five-week-old plants, which were either incubated for 12 h in the dark (dark) or for 3-4h at 80-100 µmol m^{-2} s^{-1} (light). Proteins were extracted, normalized to leaf fresh weight and loaded onto SDS-PA gels. After transfer onto PVDF membranes, PGR5 and PGRL2 were immunodetected and band intensities were quantified as described above. Immunodetection assays were carried out in three replicates and quantifications are presented as a dot plot (WT = 100%).

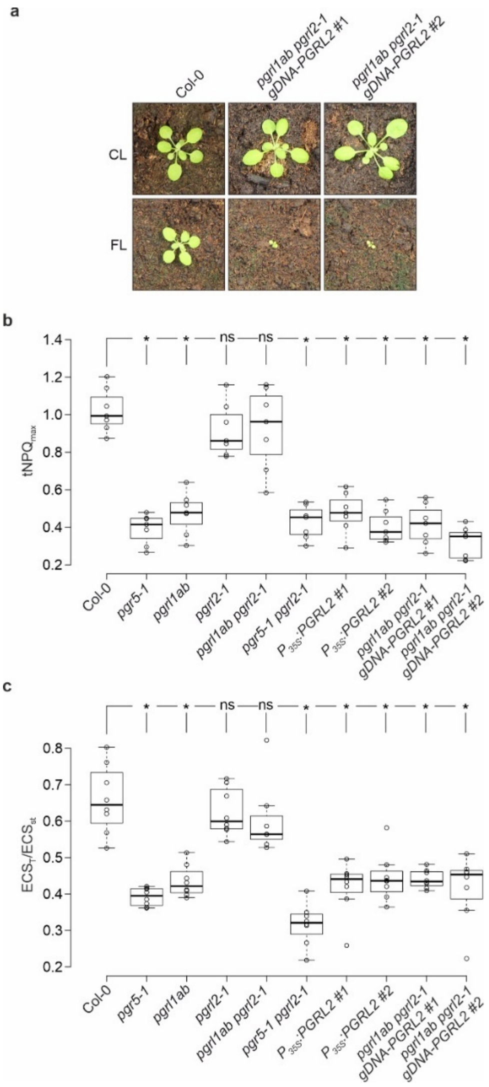

Supplementary Figure 3 Characterization of PGRL2: phenotype of complemented lines and effects of its absence or overexpression on CEF activity.
a The *pgrl1ab* phenotype is restored by introduction of the WT *PGRL2* gene into the *pgrl1ab pgrl2-1* background. Growth phenotype of *pgrl1ab pgrl2-1 gDNA-PGRL2* plants under constant (CL) and fluctuating light (FL) conditions. Two independent lines (#1 and #2) were propagated for further analyses. **b** The two complemented lines *pgrl1ab pgrl2-1 gDNA-PGRL2* #1 and #2 exhibit the same NPQ phenotype as *pgrl1ab* plants. Transient maximal NPQ (tNPQ$_{max}$) values were measured in dark-light induction experiments with a Dual-KLAS/NIR system (Walz GmbH, Effeltrich, Germany) as described in Fig 3b. Data points are shown as open circles (n = 7). **c** Electrochromic shift measurements were carried out as in Fig 3c. Both *pgrl1ab pgrl2-1 gDNA-PGRL2* lines display a reduced proton motive force at 340 µmol m^{-2} s^{-1}, which is indicated by lower ECS$_T$/ECS$_{st}$ values. Data points are shown as open circles (n = 8, 8, 8, 8, 7, 8, 8, 8). Note that the data for control lines are also shown in **Fig. 3b** and **c**. The horizontal lines in panel **b** and **c** represent the median and boxes indicate the 25th and 75th percentiles. Whiskers extend 1.5× the interquartile range, outliers are represented as dots. For statistical analyses in panel **b** and **c**, the non-parametric Kruskal-Wallis test was performed, followed by pairwise Dunn's tests. The *p*-values were adjusted on an experiment level using the Benjamini-Hochberg method. Statistically significant differences are marked with asterisks (*p ≤ 0.05, ns, not statistically significant). Exact *p*-values are provided in the Source Data file.

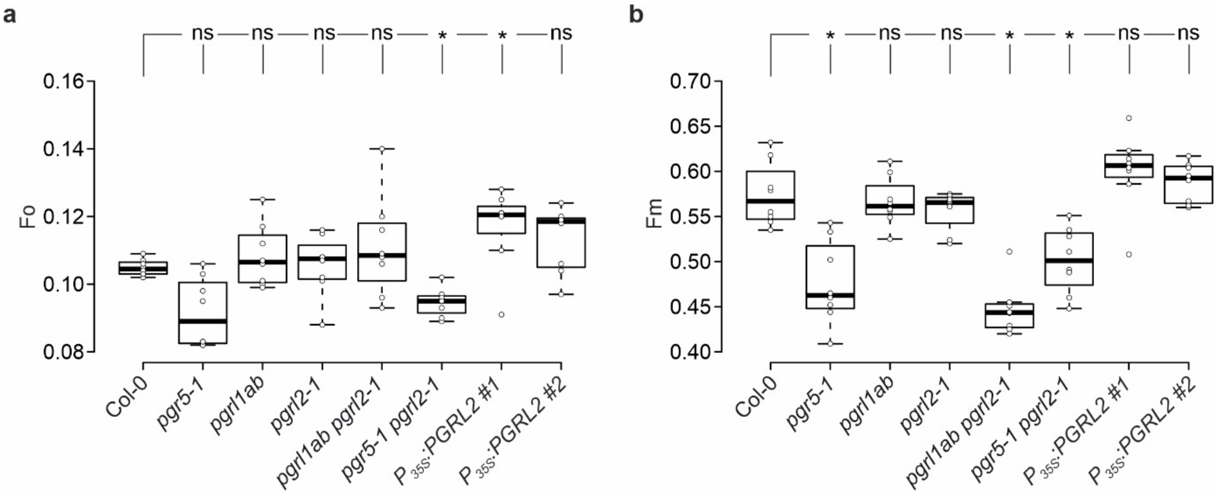

Supplementary Figure 4 Dark fluorescence yield (Fo)(**a**) and maximal fluorescence yield (Fm)(**b**) assessed before induction/recovery experiments at low light (13 µmol photons m^{-2} s^{-1}).

Plants were dark-adapted for 30 min before measurements with a Dual/KLAS-NIR spectrophotometer (Walz, Effeltrich, Germany). Data points of 8 replicates are shown. The horizontal lines represent the median and boxes indicate the 25th and 75th percentiles. Whiskers extend 1.5× the interquartile range, outliers are represented as dots. For statistical analyses, the non-parametric Kruskal-Wallis test was performed, followed by pairwise Dunn's tests. The *p*-values were adjusted on an experiment level using the Benjamini-Hochberg method. Statistically significant differences are marked with asterisks (*p ≤ 0.05, ns, not statistically significant). Exact *p*-values are provided in the Source Data file.

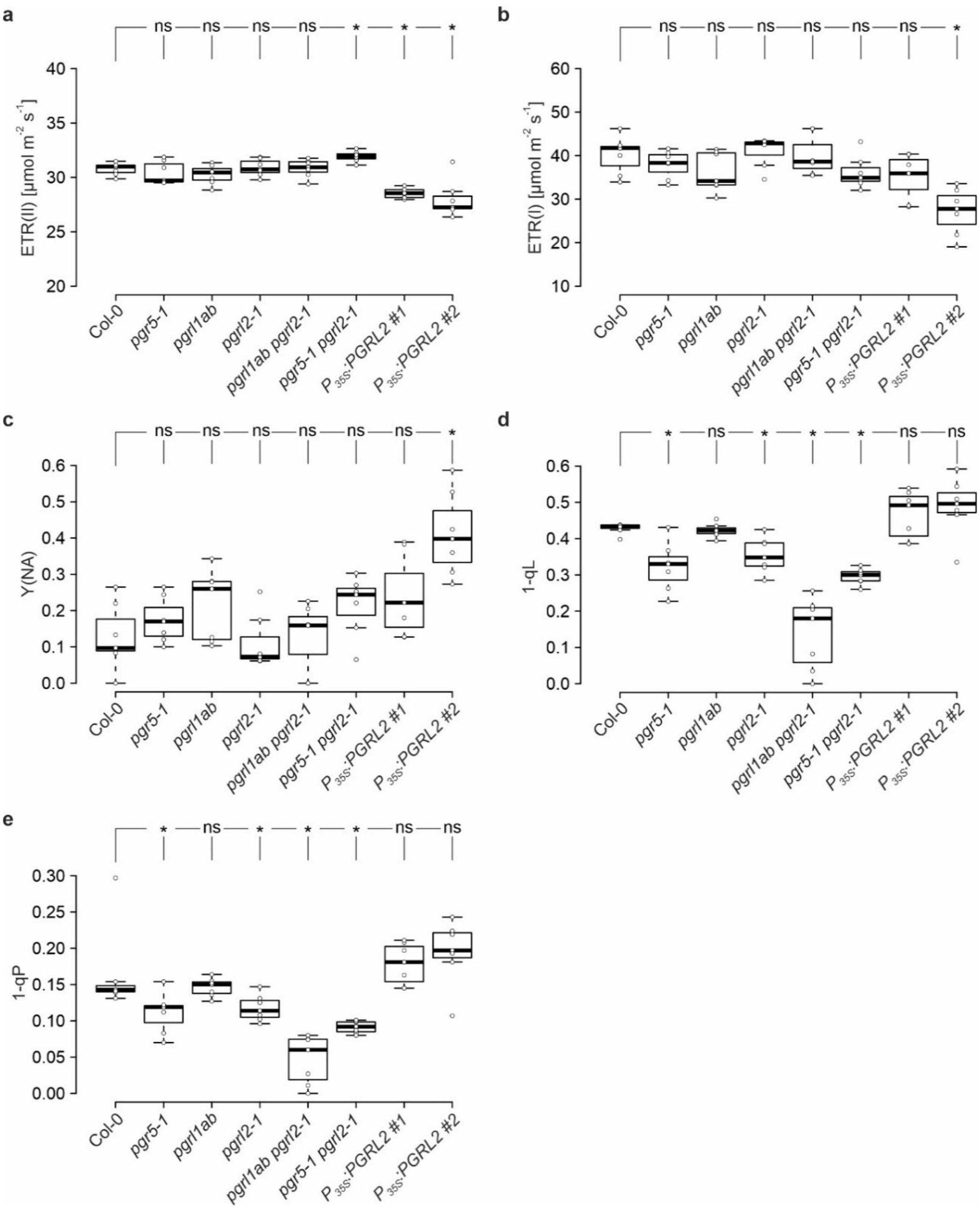

Supplementary Figure 5 Steady-state photosynthetic parameters at moderate light intensities (110 µmol photons m^{-2} s^{-1}) determined in light induction/recovery experiments.

a Electron transport rates through PSII [ETR(II)]. **b** Electron transport rates through PSI [ETR(I)]. **c** Acceptor site limitation of PSI [Y(NA)]. **d** and **e** Reduction state of the plastoquinone pool determined as 1-qL (**d**) and 1-qP (**e**). Data points of 7 replicates are shown. The horizontal lines represent the median and boxes indicate the 25th and 75th percentiles. Whiskers extend 1.5× the interquartile range, outliers are represented as dots. For statistical analyses, the non-parametric Kruskal-Wallis test was performed, followed by pairwise Dunn's tests. The *p*-values were adjusted on an experiment level using the Benjamini-Hochberg method. Statistically significant differences are marked with asterisks (*$p \leq 0.05$, ns, not statistically significant). Exact *p*-values are provided in the Source Data file.

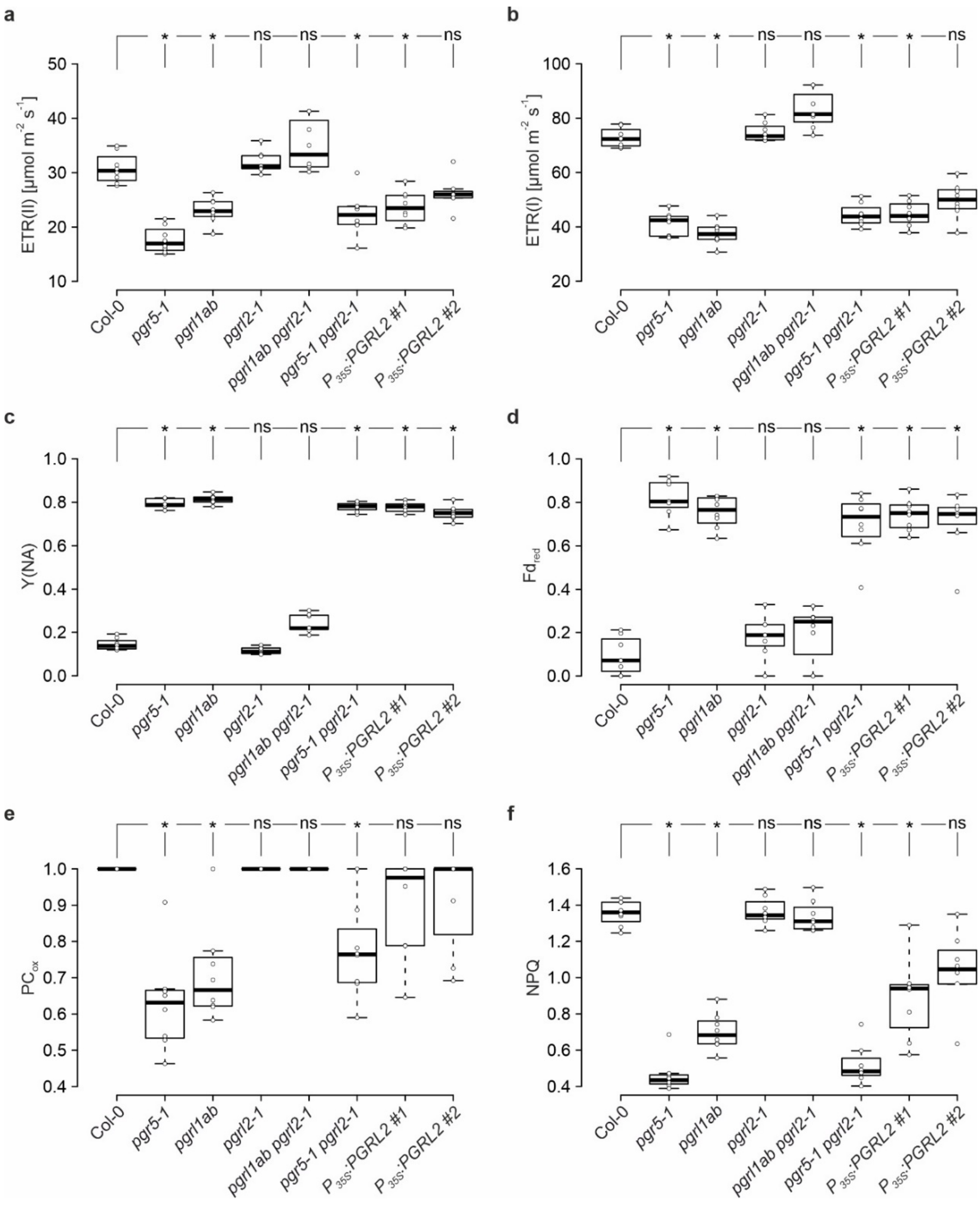

Supplementary Figure 6 Steady-state photosynthetic parameters at high light intensities (477 µmol photons m^{-2} s^{-1}) determined in light induction/recovery experiments.
a Electron transport rates through PSII [ETR(II)]. **b** Electron transport rates through PSI [ETR(I)]. **c** Acceptor site limitation of PSI [Y(NA)]. **d** and **e** Fraction of reduced ferredoxin (Fd_{red})(**d**) and oxidized plastocyanin (PC_{ox})(**e**). **f** Non-photochemical quenching (NPQ). Data points of 8 replicates are shown. The horizontal lines represent the median and boxes indicate the 25th and 75th percentiles. Whiskers extend 1.5× the interquartile range, outliers are represented as dots. For statistical analyses, the non-parametric Kruskal-Wallis test was performed, followed by pairwise Dunn's tests. The *p*-values were adjusted on an experiment level using the Benjamini-Hochberg method. Statistically significant differences are marked with asterisks (*$p \leq 0.05$, ns, not statistically significant). Exact *p*-values are provided in the Source Data file.

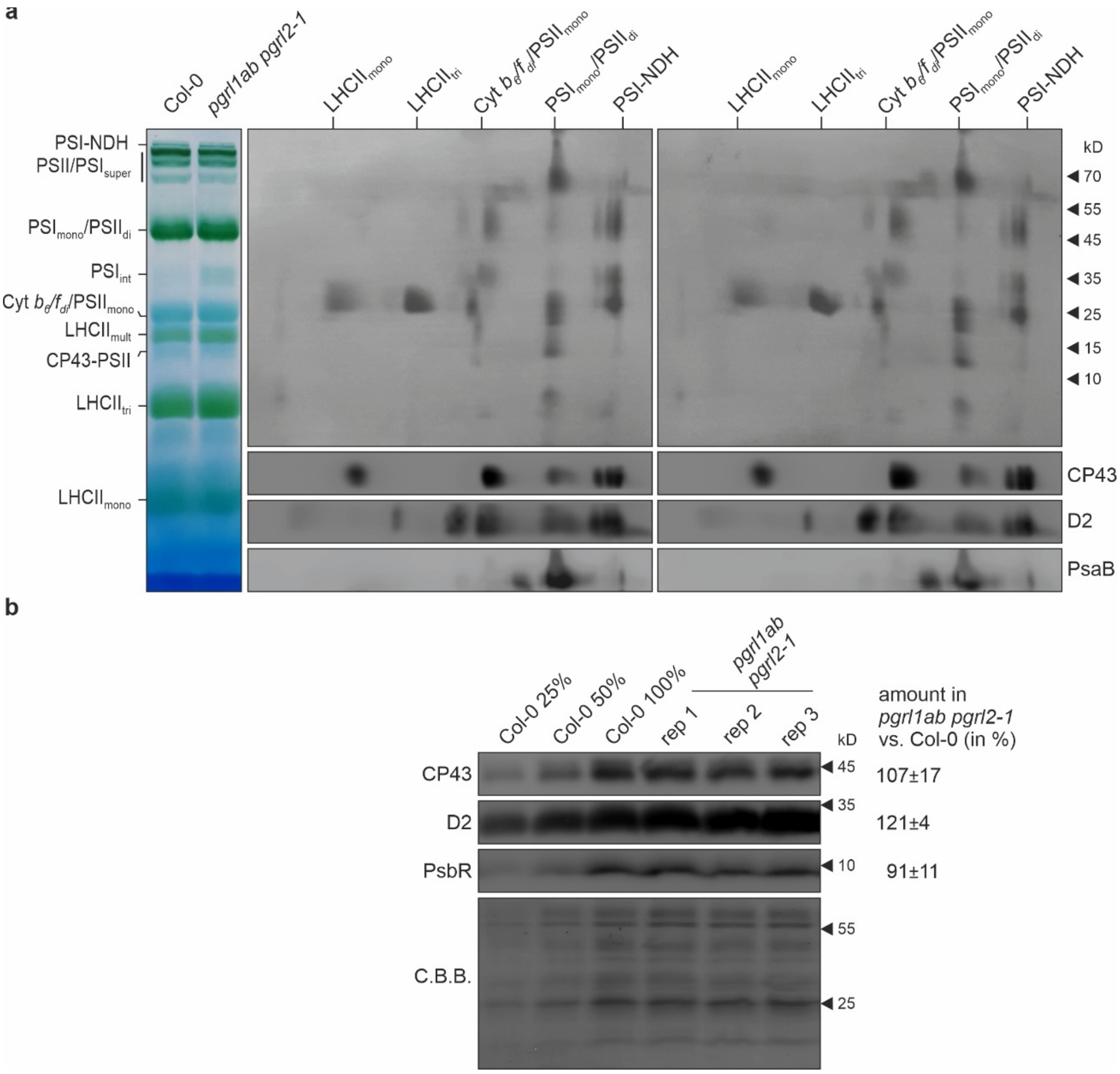

Supplementary Figure 7 PSII integrity in *pgrl1ab pgrl2-1*.

a BN-PAGE and subsequent immunodetection analyses of *pgrl1ab pgrl2-1* thylakoid protein complexes were carried out as described[1]. In brief, thylakoid membranes were isolated from dark-adapted (12h), 5-week-old wild-type and *pgrl1ab pgrl2-1* plants. After solubilization with 1% [w/v]) n-dodecyl *β*-D-maltoside, thylakoid protein complexes were fractionated by BN-PAGE (5-12% [v/v] acrylamide) and subsequently subjected to Tricine-SDS-PA (10% [v/v] acrylamide) gel electrophoresis. Separated complexes were transferred onto PVDF membranes, proteins were visualized by Coomassie Brilliant Blue G-250 staining and CP43, D2 and PsaB were immunodetected. Complex annotation was carried out as described[2]: PSI-NAD(P)H-like dehydrogenase supercomplex (PSI-NDH), PSII/PSI supercomplexes ($PSII/PSI_{super}$), PSI monomers and PSII dimers ($PSI_{mono}/PSII_{di}$), dimeric Cyt *b_6f* and PSII monomers (Cyt $b_6/f_{di}/PSII_{mono}$ and), multimeric LHCII ($LHCII_{mult}$), CP43-free PSII monomers (CP43-PSII), trimeric LHCII ($LHCII_{tri}$), and monomeric LHCII ($LHCII_{mono}$). A tentative PSI intermediate[3] is abbreviated as PSI_{int}. **b** Quantification of selected PSII marker subunits in *pgrl1ab pgrl2-1*. Thylakoid proteins corresponding to 1 µg of total Chl were separated by 10% Tricine-SDS-PAGE and transferred onto PVDF membranes. Membranes were decorated with CP43-, D2- and PSBR-specific antibodies and immunodetected signals were quantified using the Bio-1D software (Vilber Lourmat, Eberhardzell, Germany). PSII marker subunit amounts were referred to wild-type levels (in %) and averages ± standard deviations calculated from three biological replicates are indicated. Coomassie Brilliant Blue G-250 staining of PVDF membranes is presented as loading control.

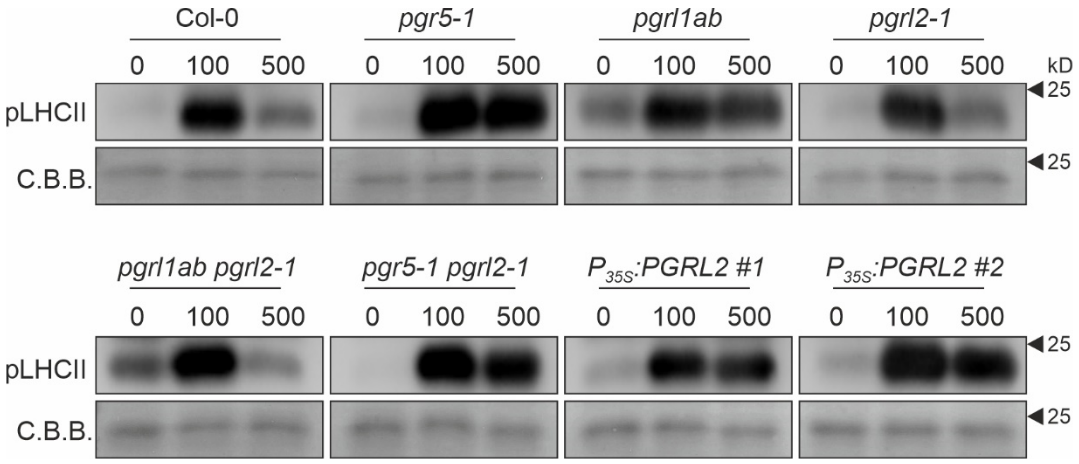

Supplementary Figure 8 LHCII phosphorylation in *pgr* mutants. Plants of Col-0, *pgr5-1*, *pgrl1ab*, *pgrl2-1*, *pgrl1ab pgrl2-1*, *pgr5-1 pgrl2-1* and the two *PGRL2* overexpressor lines (*P35S:PGRL2* #1 and #2) were kept either for 12 h in the dark (0 µmol photons m^{-2} s^{-1}), 2 h at 100 µmol photons m^{-2} s^{-1} or 2 h at 500 µmol photons m^{-2} s^{-1}. Thylakoids were isolated as described[4] in the presence of the phosphatase inhibitor NaF (10 mM), adjusted to 1 µg total chlorophyll and subjected to SDS-PAGE and immunodetection analyses with a phospho-threonine/tyrosine antibody (No. #9381, Cell Signaling Technology, Massachusetts, USA). PVDF membrane staining with Coomassie Brilliant Blue G-250 is provided as loading control (C.B.B.). Representative images out of three independent experiments are shown.

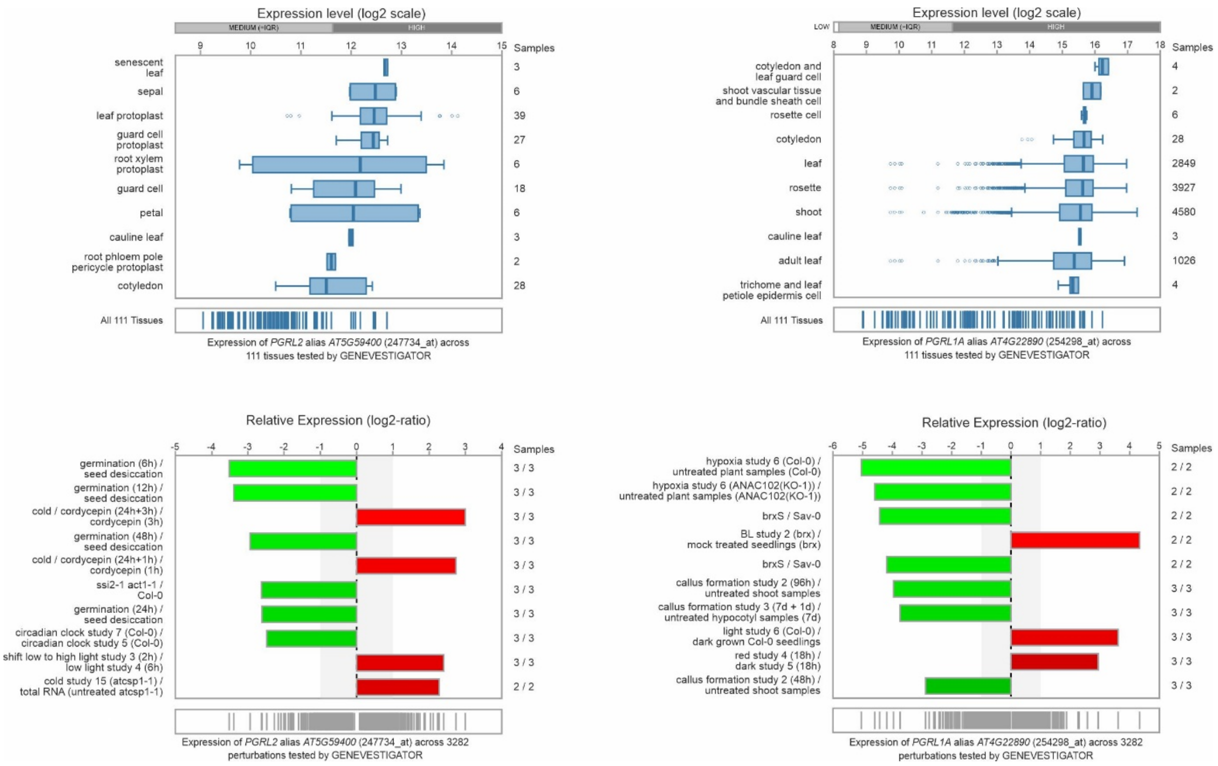

Supplementary Figure 9 Expression profiles of *PGRL2* and *PGRL1A* transcripts.
Expression profiles of transcripts of *PGRL2* (*AT5G59400*) and the major *PGRL1* isoform *PGRL1A* (*AT4G22890*). Expression levels of *PGRL2* and *PGRL1A* in different tissues and in response to diverse perturbations were obtained from Genevisible (https://genevisible.com/search) and are shown in the upper and lower panels. The vertical lines in the upper panels represent the median and boxes indicate the 25th and 75th percentiles. Whiskers extend 1.5× the interquartile range, outliers are represented as dots.

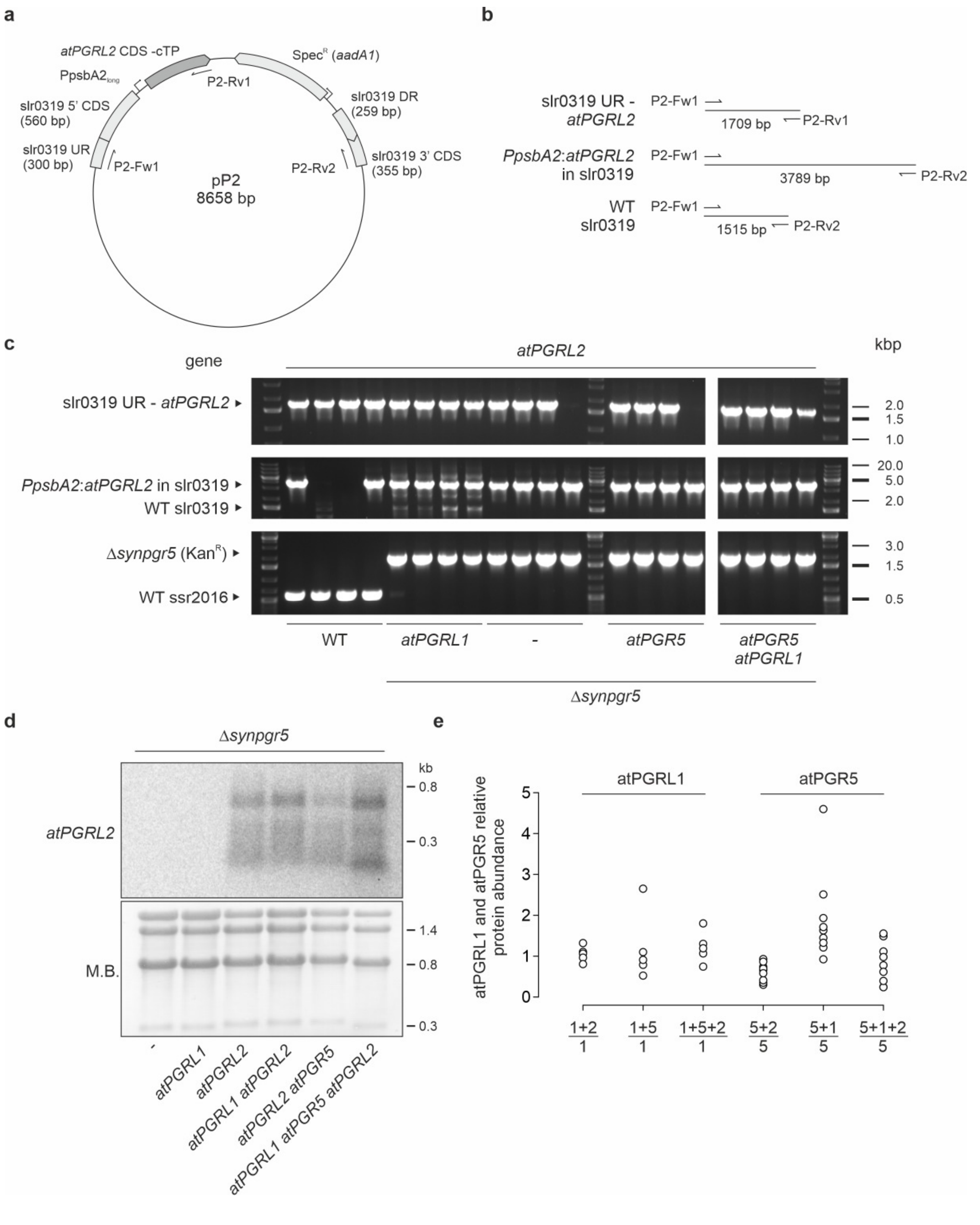

Supplementary Figure 10 Generation, genotyping and characterization of *PGRL2* expression strains.

a Genomic knock-in vector pP2 used to generate the *PGRL2* expression strain. A construct containing a P_{psbA2}:*PGRL2* gene and a spectinomycin resistance (*Spec*^{R}) cassette was spliced into the genomic *slr0319* ORF encoding β-lactamase (BlaOXA-3) by homologous recombination. UR, genomic 5' upstream region; DR, genomic 3' downstream region. Primer-binding sites used for genotyping are indicated. **b** Expected amplicon sizes and origins used for genotyping PCR. **c** Genotyping PCR of four independent transformants each. Positive clones were used for subsequent experiments. Primer-binding sites and expected amplicon sizes for the *synpgr5* mutant background were described previously[5]. **d** Accumulation of *PGRL2* transcripts. Northern analysis was performed as described[5]. **e** Quantification of effects of PGRL2 on PGR5 protein accumulation shown in **Fig. 6c.** PGRL1 and PGR5 protein accumulation in 40 µg of total membrane protein of strains expressing combinations of atPGRL1 (“1”), atPGR5 (“5”), and atPGRL2 (“2”) was quantified by immunodetection and is presented relative to atPGRL1-only (“1”) and atPGR5-only (“5”) expression strains.

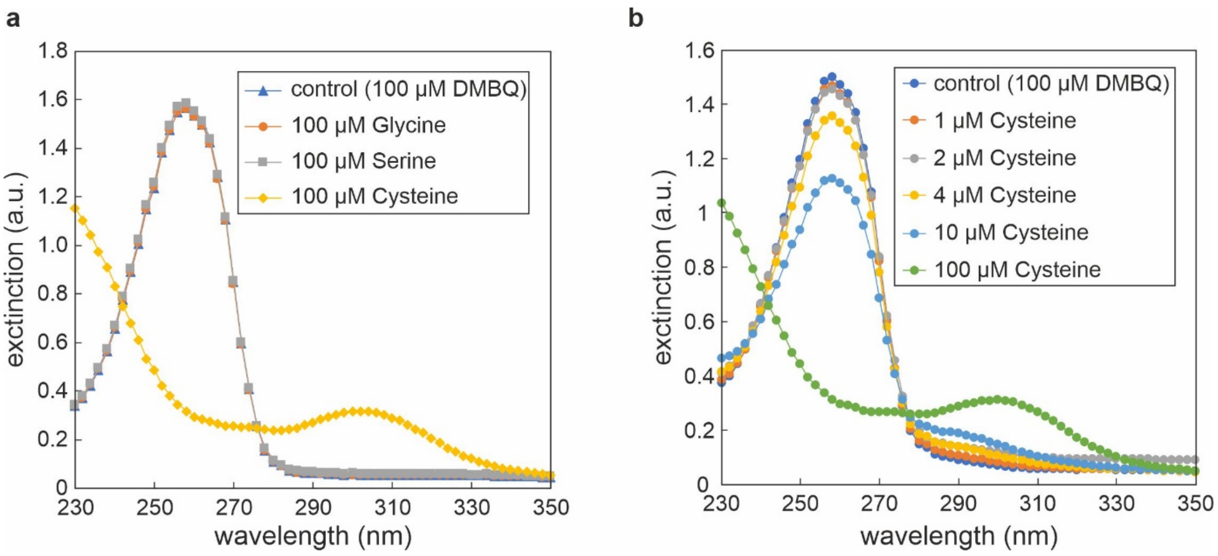

Supplementary Figure 11 Thiol addition of 2,6 dimethyl-1,4-benzoquinone (DMBQ) to cysteines.

a DMBQ (100 µM) was incubated with either 100 µM glycine, serine or cysteine for 15 min at room temperature. Extinction spectra from 230 to 350 nm were recorded with a microplate reader (Tecan Sunrise, Vienna, Austria). Averages of three technical replicates are shown. **b** DMBQ extinction changes at 260 nm depend on the DMBQ/cysteine molar ratio. Different concentrations of cysteine (1, 2, 4, 10 and 100 µM) were incubated with 100 µM DMBQ for 15 min at room temperature and subsequently analyzed as described above. We concluded that absorption at 260 nm of DMBQ can be decreased by cysteines alone via thiol addition.

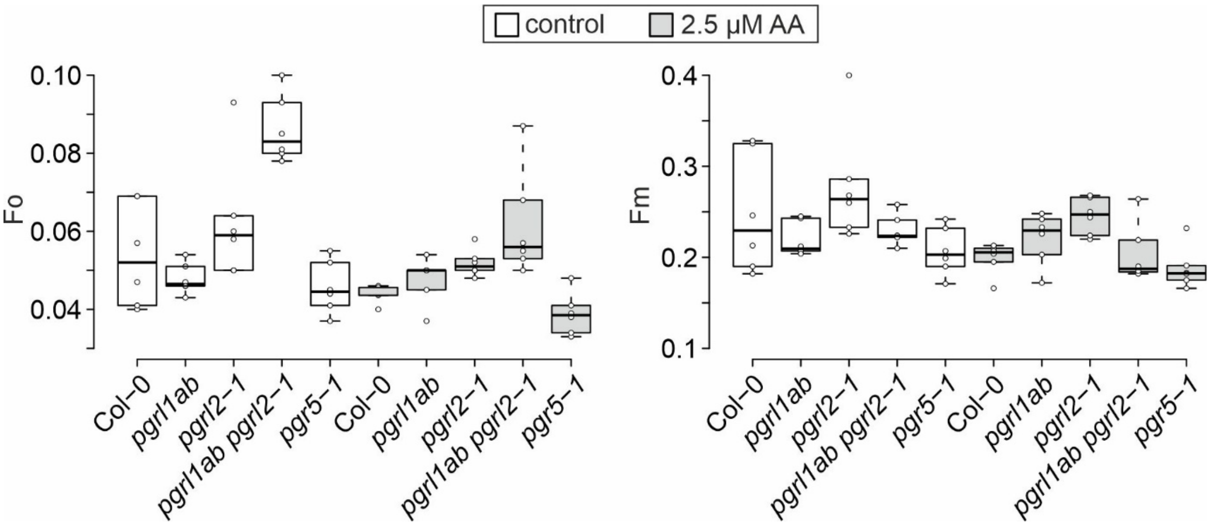

Supplementary Figure 12 Dark fluorescence (Fo) and maximal fluorescence yield (Fm) assessed in Antimycin A (AA) infiltration assays shown in Fig. 7. Detached leaves treated with either infiltration medium alone (control) or supplemented with 2.5 µM AA were dark-adapted for 5 min prior to measurements with an Imaging-PAM system (Walz, Effeltrich, Germany). Data points of 6 replicates are shown. The horizontal lines represent the median and boxes indicate the 25th and 75th percentiles. Whiskers extend 1.5× the interquartile range, outliers are represented as dots.

Supplementary Methods

BN/SDS-PAGE of thylakoid protein complexes

BN/SDS-PAGE, protein transfer onto PVDF membranes and subsequent immunodetection analyses were carried out as described[1]. Primary antibodies against CP43 (AS11 1787; 1/3,000 antibody dilution), D2 (AS06 146; 1/5,000 antibody dilution), PsbR (AS05 059; 1/15,000 antibody dilution) and PsaB (AS10 695; 1/3,000 antibody dilution) were employed according to the supplier's instructions (Agrisera AB, Vännäs, Sweden).

Determination of LHCII phosphorylation

The degree of LHCII phosphorylation was examined as described[4]. In brief, thylakoids were isolated in the presence of the phosphatase inhibitor NaF from plants either kept 12 h in the dark, 2 h in 100 or 2 h in 500 µmol photons m^{-2} s^{-1}. Thylakoid proteins corresponding to 1 µg Chl were fractionated on Tricine-SDS-PAGE (10%) and transferred to PVDF membranes. Blocking and antibody treatments were carried out in the presence of 3% [w/v] BSA. The phospho-threonine/tyrosine antibody (No. #9381) was obtained from Cell Signaling Technology (Massachusetts, USA) and used in a dilution of 1/2,500.

2,6 dimethyl-1,4-benzoquinone (DMBQ) to cysteines

Stock solutions (5 mM) of 2,6 dimethyl-1,4-benzoquinone (DMBQ) were freshly prepared in degassed ddH_2O before each assay. DMBQ (100 µM) was supplemented either with glycine (100 µM), serine (100 µM) or dilutions of cysteine (1, 2, 4, 10 and 100 µM) in a total reaction volume of 250 µL. After assays had been incubated in UV-transparent microtiter plates for 15 min, spectra (230-350 nm) were recorded with a microplate reader (Safire[2] Tecan, Männedorf, Switzerland).

Supplementary Table 1 Sequence identifiers used for the sequence comparison shown in **Fig. 1** and the phylogenetic tree in **Supplementary Fig. 1**.

No	Organism	Name in tree	identifier
1	*Chlamydomonas reinhardtii*	CrPGRL1	XP_001692513
2	*Chlorella sp.*	CsPGRL1A	XP_005850458
3	*Chlorella sp.*	CsPGRL1B	XP_005844699
4	*Volvox carteri*	VcPGRL1	XP_002951714
5	*Micromonas pusilla CCMP1545*	MpPGRL1	XP_003061604
6	*Micromonas pusilla CCMP1545*	MpPGRL2	XP_003057673
7	*Micromonas sp. RCC299*	MsPGRL1	XP_002509148
8	*Micromonas sp. RCC299*	MsPGRL2	XP_002501860
9	*Ostreococcus lucimarinus*	OlPGRL1	XP_001420406
10	*Ostreococcus lucimarinus*	OlPGRL2	XP_001417495
11	*Ostreococcus tauri*	OtPGRL1	XP_022839886
12	*Cyanidioschyzon merolae*	CmPGRL1A	XP_005539297
13	*Cyanidioschyzon merolae*	CmPGRL1B	XP_005538888
14	*Emiliania huxleyi*	EhPGRL1	XP_005787281
15	*Phaeodactylum tricornutum*	PtPGRL1	XP_002177079
16	*Thalassiosira pseudonana*	TpPGRL1	XP_002289386
17	*Arabidopsis thaliana*	AtPGRL1A	Q8H112
18	*Arabidopsis thaliana*	AtPGRL1B	AAO63369
19	*Arabidopsis thaliana*	AtPGRL2	NP_851220
20	*Oryza sativa Indica Group*	OsPGRL1	EEC83915
21	*Oryza sativa Indica Group*	OsPGRL2	EEC78386
22	*Oryza sativa Japonica cultivar-group*	OsJPGRL1	NP_001051955
23	*Physcomitrella patens subsp. patens*	PpPGRL1	XP_024386479
24	*Physcomitrella patens subsp. patens*	PpPGRL2	PNR40655
25	*Picea sitchensis*	PsPGRL1	ABK25031
26	*Populus trichocarpa*	PtrPGRL1	XP_002299573
27	*Populus trichocarpa*	PtrPGRL2	XP_002299869
28	*Sorghum bicolor*	SbPGRL1A	XP_021309274
29	*Sorghum bicolor*	SbPGRL1B	XP_021319464
30	*Sorghum bicolor*	SbPGRL2	XP_002439135
31	*Vitis vinifera*	VvPGRL1	XP_002282120
32	*Vitis vinifera*	VvPGRL2	XP_002280802
33	*Zea mays*	ZmPGRL1A	NP_001141772
34	*Zea mays*	ZmPGRL1B	XP_008652279
35	*Zea mays*	ZmPGRL2	XP_020397989
36	*Malus domestica*	MdoPGRL1	RXH95437
37	*Malus domestica*	MdPGRL2	XP_008357128
38	*Nicotiana attenuata*	NaPGRL1A	XP_019262345
39	*Nicotiana attenuata*	NaPGRL1B	XP_019225668
40	*Nicotiana attenuata*	NaPGRL2	XP_019247594

Supplementary Table 2 Oligonucleotides used as primers in this study.

primer name	sequence	purpose
PGRL2-1_RP	TGCTGATGCAGAGGTACAATG	genotyping of pgrl2-1
PGRL2-1_LP	TGTAAACTTTGAACGCCGATC	genotyping of pgrl2-1
PGRL1A_RP	CCAAAGAAGGAGGTGTTTTCC	genotyping of pgrl1a
PGRL1A_LP	CAAGAGTTTCTCCAAGCGTTG	genotyping of pgrl1a
PGRL1B_RP	GTTTGGGAACACAGTGGCTT	genotyping of pgrl1b
PGRL1B_LP	ATCAAGGAGGTCCACAAGTCT	genotyping of pgrl1b
PGR5_for	AGGTGATCACTGAGTTTTGC	genotyping of pgr5-1
PGR5_rev	TTCTTTTTAAGACCTAAGCA	genotyping of pgr5-1
LBb1_3	ATTTTGCCGATTTCGGAAC	T-DNA left border primer for SALK lines
LB3	TAGCATCTGAATTTCATAACCAATCTCCAT	T-DNA left border primer for SAIL lines
PGRL2_RT1_FOR	GCCTCGTCTCATTGGCTCTT	PGRL2 Northern probe 1
PGRL2_RT1_REV	TCAACTATCAAAGGCTTGCCA	PGRL2 Northern probe 1
PGRL2_RT2_FOR	AAGGAGACTGCCCGAATTGC	PGRL2 Northern probe 2
PGRL2_RT2_REV	TCTCTCCTTGGCCGTGAAAC	PGRL2 Northern probe 2
pAMBV4-PGRL2_for_v2	GGTCCTGCTAGCcgATGGCCCTCCACGGTGGTGGTTTAA	cloning of PGRL2 into pAMBV4 for split-ubiquitin assays
pAMBV4-PGRL2_rev_v2	GGTCCTCCATGGTCTGTAAACTTTGAACGCCGATCTCTCC	cloning of PGRL2 into pAMBV4 for split-ubiquitin assays
pADSL-PGRL2_for_v2	GGTCCTGGATCCGCCCTCCACGGTGGTGGTTTAA	cloning of PGRL2 into pADSL for split-ubiquitin assays
pADSL-PGRL2_rev_v2	GGTCCTGAATTCcgTTATGTAAACTTTGAACGCCGATCTCT	cloning of PGRL2 into pADSL for split-ubiquitin assays
pADSL-PGRL1A_for_v2	GGTCCTGGATCCGCCACAACAGAGCAATCAGG	cloning of PGRL1A into pADSL for split-ubiquitin assays
pADSL-PGRL1A_rev_v2	GGTCCTGAATTCcgTTAAGCTTGGCTTCCTTCTG	cloning of PGRL1A into pADSL for split-ubiquitin assays
oePGRL2_GW_for	GGGGACAAGTTTGTACAAAAAAGCAGGCTCAATGGCGGGTACTTGCACTTC	Gateway cloning of PGRL2 CDS into pDONR207 (for pH2GW7)
oePGRL2_GW_rev	GGGGACCACTTTGTACAAGAAAGCTGGGTTTATGTAAACTTTGAACGCCGATC	Gateway cloning of PGRL2 CDS into pDONR207 (for pH2GW7)
oePGRL2-eGFP_GW_rev	GGGGACCACTTTGTACAAGAAAGCTGGGTATGTAAACTTTGAACGCCGATC	Gateway cloning of PGRL2 CDS into pDONR207 (for pB7FWG2)
genomicPGRL2_GW_for	GGGGACAAGTTTGTACAAAAAAGCAGGCTCAAATACATTTAGCAACGACATCG	Gateway cloning of PGRL2 into pDONR207 (for pHGW)

genomicPGRL2_GW_rev	GGGGACCACTTTGTACAAGAAAGCTGGGTAAAAAAAGCTGAGCAACATTCT	Gateway cloning of PGRL2 into pDONR207 (for pHGW)
PGRL2_seq_for	GATGATGCATCGCAAGTTCTC	sequencing primer for PGRL2 constructs
PGRL2_seq_rev	CTGCAGAAGATGCAATCGG	sequencing primer for PGRL2 constructs
MBP_PGRL1C_for	GCCCTCCACGGTGG	cloning of PGRL2(41-137 aa) CDS into pMal-c5x
MBP_PGRL1C-N_rev	ggtcctgaattcttaGAGAACTTGCGATGCATCAT	cloning of PGRL2(41-137 aa) CDS into pMal-c5x
pET151_PGRL2-N_for	CACCGCCCTCCACGGTGG	cloning of PGRL2(41-137 aa) CDS into pET151
pET151_PGRL2-N_rev	TTAGAGAACTTGCGATGCATCAT	cloning of PGRL2(41-137 aa) CDS into pET151
Backbone_for	ATCCGTTTTGCCCACCTGGGCCCACTGCATC	Gibson assembly of PGRL2 expression construct from Synechocystis slr0319
Backbone_rev	GGGTTCGCTCTATGCTTACCTGTTTAAACTATCAGTGTTTGACAG	Gibson assembly of PGRL2 expression construct from Synechocystis slr0319
slr0319 UR_for	AAACAGGTAAGCATAGAGCGAACCCTGGCG	Gibson assembly of PGRL2 expression construct from Synechocystis slr0319
slr0319 UR_rev	TTTTTTCCATGGGGTACTTGAGTCTACATTCCCGTAG	Gibson assembly of PGRL2 expression construct from Synechocystis slr0319
PpsbA2_for	AGACTCAAGTACCCCATGGAAAAAACGACAATTAC	Gibson assembly of PGRL2 expression construct from Synechocystis slr0319
PpsbA2_rev	GTGGAGGGCCATTTGGTTATAATTCCTTATGTATTTGTC	Gibson assembly of PGRL2 expression construct from Synechocystis slr0319
AtPgrl2_for	GAATTATAACCAAATGGCCCTCCACGGTGGT	Gibson assembly of PGRL2 expression construct from Synechocystis slr0319
AtPgrl2_rev	GAATTGAGCGGCCTACACAAGTTCATCAAACTCAAATGTGACGAG	Gibson assembly of PGRL2 expression construct from Synechocystis slr0319
SpecR RC_for	TGAACTTGTGTAGGCCGCTCAATTCGCTGCG	Gibson assembly of PGRL2 expression construct from Synechocystis slr0319
SpecR RC_rev	AGCACCTTTGCTACTCTAGAAGAACAGCAAGGCCG	Gibson assembly of PGRL2 expression construct from Synechocystis slr0319
slr0319 DR_for	GTTCTTCTAGAGTAGCAAAGGTGCTTATTG	Gibson assembly of PGRL2 expression construct from Synechocystis slr0319
slr0319 DR_rev	GTGGGCCCAGGTGGGCAAAACGGATTTAGAG	Gibson assembly of PGRL2 expression construct from Synechocystis slr0319

Supplementary References

1. Rühle, T., *et al.* The Arabidopsis protein CONSERVED ONLY IN THE GREEN LINEAGE160 promotes the assembly of the membranous part of the chloroplast ATP synthase. *Plant Physiol* **165**, 207-226 (2014).

2. Armbruster, U., *et al.* The photosynthesis affected mutant68-like protein evolved from a PSII assembly factor to mediate assembly of the chloroplast NAD(P)H dehydrogenase complex in Arabidopsis. *Plant Cell* **25**, 3926-3943 (2013).

3. Wittenberg, G., *et al.* Identification and characterization of a stable intermediate in photosystem I assembly in tobacco. *Plant J* **90**, 478-490 (2017).

4. Pribil, M., Pesaresi P., Hertle A., Barbato R. & Leister D. Role of plastid protein phosphatase TAP38 in LHCII dephosphorylation and thylakoid electron flow. *PLoS Biol* **8**, e1000288 (2010).

5. Dann, M. & Leister D. Evidence that cyanobacterial Sll1217 functions analogously to PGRL1 in enhancing PGR5-dependent cyclic electron flow. *Nat Commun* **10**, 5299 (2019).
