# Supplementary material for: PGRL2 triggers degradation of PGR5 in the absence of PGRL1
Source: Nat Commun. 2021 Jun 24;12:3941. doi: 10.1038/s41467-021-24107-7 (PMC8225790; doi:10.1038/s41467-021-24107-7)
Supplement: Supplementary file 2 — Reporting Summary [file 41467_2021_24107_MOESM2_ESM.pdf]

nature research

Corresponding author(s): Dario Leister

Last updated by author(s): May 27, 2021

Reporting Summary

Nature Research wishes to improve the reproducibility of the work that we publish. This form provides structure for consistency and transparency in reporting. For further information on Nature Research policies, see our Editorial Policies and the Editorial Policy Checklist.

Statistics

For all statistical analyses, confirm that the following items are present in the figure legend, table legend, main text, or Methods section.

n/a	Confirmed	
☐	☒	The exact sample size (*n*) for each experimental group/condition, given as a discrete number and unit of measurement
☐	☒	A statement on whether measurements were taken from distinct samples or whether the same sample was measured repeatedly
☐	☒	The statistical test(s) used AND whether they are one- or two-sided<br>*Only common tests should be described solely by name; describe more complex techniques in the Methods section.*
☒	☐	A description of all covariates tested
☐	☒	A description of any assumptions or corrections, such as tests of normality and adjustment for multiple comparisons
☐	☒	A full description of the statistical parameters including central tendency (e.g. means) or other basic estimates (e.g. regression coefficient) AND variation (e.g. standard deviation) or associated estimates of uncertainty (e.g. confidence intervals)
☐	☒	For null hypothesis testing, the test statistic (e.g. *F*, *t*, *r*) with confidence intervals, effect sizes, degrees of freedom and *P* value noted<br>*Give P values as exact values whenever suitable.*
☒	☐	For Bayesian analysis, information on the choice of priors and Markov chain Monte Carlo settings
☒	☐	For hierarchical and complex designs, identification of the appropriate level for tests and full reporting of outcomes
☒	☐	Estimates of effect sizes (e.g. Cohen's *d*, Pearson's *r*), indicating how they were calculated

Our web collection on statistics for biologists contains articles on many of the points above.

Software and code

Policy information about availability of computer code

Data collection	Dual-KLAS/NIR (V2.072, Walz GmbH, Germany, https://www.walz.com/products/chl_p700/dual-klas-nir/downloads.html)<br><br>ImagingWin (V2.41a, Walz GmbH, Germany, https://www.walz.com/products/chl_p700/imaging-pam_ms/downloads.html)<br><br>ECS, Chl a and P700 measurements (Dual-PAM-100 Software DualPAM V3.10, Walz GmbH, Germany, https://www.walz.com/products/chl_p700/dual-pam-100/downloads.html)<br><br>Immunodetection (Fusion, Version 15-18, Vilber Lourmat, Eberhardzell, Germany)<br><br>Northern analyses (Typhoon Phosphor Imager System, v5.0.1, GE Healthcare)<br><br>Extinction measurements in microtiter plates (Magellan 6, Tecan, Männedorf, Switzerland)
Data analysis	Signal quantification in immunodetection assays (Bio-1D, version 15.03, Vilber Lourmat, Eberhardzell, Germany)<br><br>Transit peptide prediction (ChloroP, http://www.cbs.dtu.dk/services/ChloroP/)<br><br>Transmembrane domain prediction (TMHMM, http://www.cbs.dtu.dk/services/TMHMM/)<br><br>Sequence alignment (Vector NTI, ThermoFisher Scientific, Waltham, Massachusetts, USA)<br><br>Sequence alignment formatting (boxshade, https://embnet.vital-it.ch/software/BOX_form.html)

Construction of the phylogenetic tree (CLC Main Workbench software, version 8.1.2, Qiagen, Venlo Netherlands)

Boxplot analyses (http://shiny.chemgrid.org/boxplotr/)

Heat map analyses (ClustVis, https://biit.cs.ut.ee/clustvis/)

Statistical analyses were carried out in R v3.5.2 (https://www.r-project.org/) using the R package dunn.test (version 1.3.5) and t.test.

Statistical analyses in Fig. 6d (https://astatsa.com/ and https://www.r-project.org/)

Gene expression analyses (https://genevisible.com/search)

For manuscripts utilizing custom algorithms or software that are central to the research but not yet described in published literature, software must be made available to editors and reviewers. We strongly encourage code deposition in a community repository (e.g. GitHub). See the Nature Research guidelines for submitting code & software for further information.

Data

Policy information about availability of data

All manuscripts must include a data availability statement. This statement should provide the following information, where applicable:

- Accession codes, unique identifiers, or web links for publicly available datasets
- A list of figures that have associated raw data
- A description of any restrictions on data availability

The authors declare that all data presented in this study are available in the figures and the accompanying Supplementary Information file. The source data underlying Figs. 2b, 2c, 3, 4, 5, 6a, 6c, 6d and 7, and Supplementary Figs 2b, 2c, 2d, 2e, 3b, 3c, 4, 5, 6, 7, 8, 10d, 10e, 11 and 12, as well as detailed corresponding statistics, are provided as a Source Data file. Other data that support the study are available from the corresponding author upon reasonable request.

Field-specific reporting

Please select the one below that is the best fit for your research. If you are not sure, read the appropriate sections before making your selection.

[x] Life sciences [ ] Behavioural & social sciences [ ] Ecological, evolutionary & environmental sciences

For a reference copy of the document with all sections, see nature.com/documents/nr-reporting-summary-flat.pdf

Life sciences study design

All studies must disclose on these points even when the disclosure is negative.

Sample size

Sample sizes were chosen based on prior experience and typical sample sizes reported in the literature.

Sample sizes in Arabidopsis studies:
9-17 plants from each genotype were selected for fresh weight determination.
7 plants from each genotype were analyzed for their maximal transient NPQ.
7-8 plants of each genotype were studied with respect to their proton motif force (pmf) under high light.
17 plants were analyzed with respect to plastoquinone reduction and P700 oxidation kinetics.
7-8 plants of each genotype were examined in light induction/recovery experiments [13, 110 and 477 µmol photons m(-2) s(-1)]
6 leaves of 6 indiviual plants were analyzed for each genotype in Antimycin A infiltration assays.

Sample sizes in Synechocystis studies:
Sample size for P700 PAM measurements was dictated by the time point at which the maximum amount of cultures simultaneously had reached a growth stage suitable for measurement, and the number of according cultures per genotype having reached/not surpassed this point. With cultivation room and media batch size being practical limiting factors, the sample sizes presented are the maximum we could obtain in the context of a single cultivation batch. The presented sample size is regarded sufficiently representative for reasons of the employed working system being prokaryotic, and performed measurements of 2 ml OD5 cells yielding integrals over the physiological state of >10^8 cells for each data point, respectively.

Data exclusions

n.a.

Replication

Arabidopsis:

Photosynthetic analyses were carried out with at least 6 independent biological replicates (6 individual plants). Imaging-PAM analysis (Antimycin A infiltration assays) were conducted with 3-4 week-old Arabidopsis plants. All photosynthetic measurements were conducted with 5-6 week-old Arabidopsis plants (12/12h dark/light cycle).

Synechocystis:
Subsets of the entire mutant set presented were measured in the same manner as described repeatedly before the presented study. Qualitatively, results were found to be highly robust. However, quantitative deviations of P700 oxidation half times of identical genotypes/

clones were observed among batches, probably resulting from a mixture of (i) slight inconsistencies between media batches, (ii) differences in age/vitality of plate cultures used as inoculum, and, apparently, (ii) outside climate. Hence, we decided to acquire to-be-shown data from a single batch of media and antibiotics, and cultivate all assayed cell material simultaneously to optimize data signal-to-noise ratio.

Quantification of PGR5, PGRL1 and PGRL2:
PGR5, PGRL1 and PGRL2 amounts were quantified in at least three independent immunodetection assays with protein samples isolated from at least three different biological samples. Data are presented as dot plots throughout the whole study and averages +/- standard deviations are provided in the main text.

PSII integrity in pgrl1ab pgrl2-1 plants was examined from three independent thylakoid isolation procedures (for each isolation, 4-6 plants were pooled). Immunodetection and quantification of 3 individual PSII marker subunits (CP43, D2 and PSBR) were conducted.

LHCII phosphorylation was analyzed by immunodetection assays in at least three independent experiments with at least three independent biological replicates. For each light treatment [0, 100 and 500 µmol photons m(-2) s(-1)], 3 plants were pooled for thylakoid isolation.

Randomization

Arabidopsis:
Trays with Arabidopsis plants were randomly distributed in the growth chambers to avoid differences in light irradtion, watering and humidity.

Synechocystis:
Synechocystis cultures were distributed randomly on shakers during cultivation stage, and assigned random numbers prior to preparation for PAM measurements. Measurements were conducted in ascending order of assigned numbers, yielding randomized running order of physiological measurements.

Blinding

Arabidopsis:
Since the knockout and overexpressor lines showed clear photosynthetic phenotypes (e.g. differences in Fv/Fm, Y(NA) or NPQ), blinding experiments were not necessary.

Synechocystis:
Culture identity was only determined after measurements and subsequent protein extraction were performed, rendering further blinding unnecessary.

Reporting for specific materials, systems and methods

We require information from authors about some types of materials, experimental systems and methods used in many studies. Here, indicate whether each material, system or method listed is relevant to your study. If you are not sure if a list item applies to your research, read the appropriate section before selecting a response.

Materials & experimental systems

n/a	Involved in the study	
☐	☒	Antibodies
☒	☐	Eukaryotic cell lines
☒	☐	Palaeontology and archaeology
☒	☐	Animals and other organisms
☒	☐	Human research participants
☒	☐	Clinical data
☒	☐	Dual use research of concern

Methods

n/a	Involved in the study	
☒	☐	ChIP-seq
☒	☐	Flow cytometry
☒	☐	MRI-based neuroimaging

Antibodies

Antibodies used

Anti-PGR5, provided by Prof. T. Shikanai, used dilution 1/2,500
Anti-PGRL1, generation described in DalCorso et al. (2008), used dilution 1/10,000
Anti-PGRL2, generated in this study by the antibody service company Pineda (Berlin, Germany), used dilution 1/2,000
Anti-PetA, Agrisera, AS08 306, lot number 1312, used dilution 1/5,000
Anti-CSP41b, provided by David Stern, used dilution 1/5,000
Anti-CP43, Agrisera, AS11 1787, lot number 1609, used dilution 1/3,000
Anti-D2, Agrisera, AS06 146, lot number 1706, used dilution 1/5,000
Anti-PSBR, Agrisera, AS05 059, lot number not provided, used dilution 1/15,000
Anti-PsaB, Agrisera, AS10 695, lot number 1603, used dilution 1/3,000
Anti-phospho-threonine/tyrosine antibody, Cell Signaling Technology, No. #9381, lot number 25, used dilution 1/2,500
Goat Anti-Rabbit IgG Antibody HRP-conjugate (Sigma-Aldrich A9169), batch number 015M4858V, used dilution 1/30,000

Validation

- Anti-PGR5 antibodies were raised in rabbits against the peptide sequence ADAKQRQGLIRLAKKNGERL and validation is described in Munekage et al. [Munekage, Y. et al. PGR5 Is Involved in Cyclic Electron Flow around Photosystem I and Is Essential for Photoprotection in Arabidopsis. Cell 110, 361–371 (2002)].

- Generation and validation of Anti-PGRL1 has been described by DalCorso et al. [DalCorso, G. et al. A complex containing PGRL1 and PGR5 is involved in the switch between linear and cyclic electron flow in Arabidopsis. Cell 132, 273–85 (2008)].

- Anti-PGRL2 was generated in this study as described in the Methods section. In brief, the N-terminal sequence of PGRL2 (41-137 AA) fused to a maltose-binding protein was heterologously expressed in and purified from E. coli cells (BL21, DE3). Antibody generation in rabbits and purification was carried out by the antibody service company Pineda (Berlin, Germany, http://www.pineda-abservice.de). Since the antibody failed to detect wild-type PGRL2 levels, it was validated with Arabidopsis lines overexpressing PGRL2 and purified, recombinant 6xHis-PGRL2(41-137AA) (Supplementary Fig. 2d).

- Anti-CSP41b was provided by David Stern [Bollenbach, T. J., Sharwood, R. E., Gutierrez, R., Lerbs-Mache, S. & Stern, D. B. The RNA-binding proteins CSP41a and CSP41b may regulate transcription and translation of chloroplast-encoded RNAs in Arabidopsis. Plant Mol. Biol. 69, 541–552 (2009)] and was also validated and employed in Qi et al. [Qi, Y. et al. Arabidopsis CSP41 proteins form multimeric complexes that bind and stabilize distinct plastid transcripts. J. Exp. Bot. 63, 1251–1270 (2012)].

- Antibodies obtained from Agrisera (https://www.agrisera.com/). Validation is provided on the following web pages:
Anti-PetA (AS08 306, https://www.agrisera.com/en/artiklar/cytochrome-f-peta-di-and-monocots.html)
Anti-CP43 (AS11 1787, https://www.agrisera.com/en/artiklar/psbc-cp43-protein-of-psii-2.html)
Anti-D2 (AS06 146, https://www.agrisera.com/en/artiklar/psbd-d2-global-antibody.html)
Anti-PSBR (AS05 059, https://www.agrisera.com/en/artiklar/psbr-10-kda-protein.html)
Anti-PsaB (AS10 695, https://www.agrisera.com/en/artiklar/psab-psi-b-core-subunit-of-photosystem-i-2.html)

- The phospho-threonine/tyrosine antibody (No. #9381) was purchased from Cell Signaling Technology (Massachusetts, USA). Validation is provided on the following web page:
https://www.cellsignal.com/products/primary-antibodies/phospho-threonine-tyrosine-antibody/9381
